# Dynamic Network Characteristics of Adolescent Mental Health Symptoms: Gender and Grade Differences Based on a Cross-Lagged Panel Network Model

**DOI:** 10.3390/bs16060928

**Published:** 2026-06-05

**Authors:** Sisi Li, Guangzhen Zhang, Zongbao Liang, Dongquan Liang

**Affiliations:** 1 Key Laboratory of Child Development and Learning Science, School of Biological Science & Medical Engineering, Southeast University, Nanjing 211189, China; 230268711@seu.edu.cn (S.L.);; 2 School of Education Science, Nanning Normal University, Nanning 530299, China

**Keywords:** adolescent mental health, Cross-Lagged Panel Network, gender differences, grade-level differences, symptom network

## Abstract

This study used a Cross-Lagged Panel Network model to examine prospective longitudinal associations among dimensions of adolescent mental health and differences in these associations across gender and grade levels. A total of 3610 Chinese adolescents completed the Middle School Student Mental Health Scale at two time points, with an interval of approximately six months between assessments. In the overall network, interpersonal sensitivity had the strongest out-expected influence, indicating the strongest outgoing predictive associations with other mental health dimensions. Depression ranked second and showed a significant bidirectional prospective association with interpersonal sensitivity. Emotional instability had the strongest in-expected influence, suggesting that it was the dimension most strongly predicted by other domains. Subgroup analyses revealed that interpersonal sensitivity showed the strongest outgoing predictive associations in the male network, whereas depression played this role in the female network. In the junior high school network, depression showed the strongest outgoing predictive associations, whereas interpersonal sensitivity was the most central predictive domain in the senior high school network. These findings may inform gender- and grade-sensitive screening and monitoring strategies and provide preliminary evidence for future intervention research.

## 1. Introduction

Adolescence is a critical transitional period from childhood to adulthood, during which individuals undergo substantial changes in physiological functioning, cognitive development, and social roles. This stage is not only crucial for the development of positive psychological capacities, such as resilience and psychological adaptability, but also a sensitive period characterized by a high incidence of mental health problems (Blakemore, 2019; Geldhof et al., 2015). In recent years, adolescent mental health has increasingly become a central concern in global public health and education (James et al., 2018; Racine et al., 2021; Wainberg et al., 2017). According to a report by the World Health Organization, approximately 13% of adolescents worldwide experience at least one mental disorder (Garaigordobil, 2023). In China, the overall mental health status of adolescents is also far from optimistic and is closely related to the internal developmental challenges they face. Adolescents must not only cope with the development of self-identity but also navigate conflicts between self-identity and external factors such as family, school, and society (Wu et al., 2023). A meta-analysis found that the prevalence rates of depression and anxiety among Chinese children and adolescents were 29% and 26%, respectively (Ma et al., 2021), highlighting the substantial psychological distress and burden experienced by this population. As a major global public health concern, adolescent mental health problems require a thorough understanding of the mechanisms underlying their onset, maintenance, and progression to inform the development of effective intervention strategies.

Mental health is a multifaceted construct that cannot be adequately characterized by any single indicator or dataset. The World Health Organization defines mental health as a state of well-being in which individuals are able to cope with the stresses of life, realize their abilities, learn and work effectively, and contribute to society (Cilar Budler & Stiglic, 2023). Research has shown that mental health problems may lead to poorer academic performance, school dropout, increased engagement in high-risk behaviors, self-harm, and even suicide (Fergusson & Woodward, 2002; Luo et al., 2020). These mental health challenges not only impose substantial emotional and psychological burdens on individuals and their families but also result in considerable social and economic costs. For example, adolescent mental health problems can lead to increased healthcare expenditures, reduced productivity, and higher social service costs (Fatori et al., 2018). In addition, many mental disorders that first emerge during adolescence may persist into adulthood if left untreated, thereby further increasing the burden of disease and associated economic costs (Pine et al., 1998; Stelmach et al., 2022).

Previous research has shown that adolescent mental health problems differ significantly by gender and grade level. With regard to gender, female adolescents tend to report higher levels of anxiety and depression, which may be closely associated with greater emotional reactivity, heightened social sensitivity, and pervasive academic pressure in Chinese society (Kirkcaldy et al., 2003; Lewinsohn et al., 1998). In contrast, male adolescents are more likely to exhibit behavioral problems and externalizing symptoms, such as impulsive and antisocial behaviors (Habersaat et al., 2018). These tendencies may be related to hormonal changes during puberty and may also be shaped by gender role expectations during socialization (Buchanan et al., 1992; Nori et al., 2009). With regard to grade level, the prevalence of anxiety is highest during junior high school. Because students at this stage are in early adolescence, their prefrontal cortex is still maturing, their emotion regulation capacity remains relatively limited, and they are highly sensitive to environmental threats, making them more likely to respond to uncertain challenges with anxiety (Zhang et al., 2022). In contrast, the prevalence of depression is highest among senior high school students. Having entered late adolescence, these students are exposed to prolonged and intense academic pressure, as well as the highly competitive environment associated with the National College Entrance Examination. The accumulation of chronic stress, together with negative cognitive rumination, may make them more vulnerable to depressive states characterized by helplessness and low mood (Yu et al., 2022).

These differences not only influence the manifestation of individual symptoms but also shape the network relationships among symptoms (Birkeland et al., 2017). From a developmental perspective, gender and grade level may alter symptom networks because adolescents in different subgroups are exposed to different combinations of biological maturation, social expectations, academic stress, and interpersonal demands (Shalayiding et al., 2024). These contextual and developmental differences may affect not only the severity of specific symptoms but also the ways in which one symptom activates, maintains, or reinforces another over time. For example, previous research has found that, among female students, depressed mood was the central symptom, and poor school adaptation was more closely associated with problems such as lack of motivation and self-devaluation. In contrast, among male students, difficulty relaxing emerged as the central symptom, and poor school adaptation was more prominently reflected in irritability and its associations with relationships with teachers and peers (Shalayiding et al., 2024). Similarly, students at different grade levels have been shown to differ in the structure of their symptom networks: among junior high school students, the core symptoms include “difficulty relaxing,” “uncontrollable worry,” and “anxiety when without a mobile phone,” whereas among senior high school students, the core symptoms are “uncontrollable worry,” “loss of interest,” and “nervousness” (Gao et al., 2025). Therefore, adolescents of different genders and grade levels may require differentiated intervention strategies when facing similar mental health problems. By identifying and addressing the unique challenges faced by male and female students, as well as by junior and senior high school students, educators and mental health professionals can develop more inclusive and effective support systems to better meet the diverse mental health needs of Chinese adolescents.

In recent years, network analysis has provided an important methodological approach for understanding the complex interrelationships among adolescent mental health symptoms (Guloksuz et al., 2017). This approach is grounded in the network theory of psychopathology proposed by Borsboom (2008), which conceptualizes mental disorders as dynamic causal systems arising from direct interactions among symptoms rather than as manifestations of a single latent disease entity. Network analysis therefore conceptualizes psychological conditions as systems composed of direct interactions among symptoms, rather than as collections of indicators driven by an underlying disorder (Sun & Zhong, 2023). In network models, nodes represent individual symptoms or risk factors, whereas edges reflect the strength of the associations between them (Hofmann et al., 2016). By calculating centrality indices, researchers can identify the most influential nodes within the network; by identifying bridge nodes, they can further reveal potential comorbidity mechanisms linking different communities within the network (Epskamp et al., 2018). According to this theoretical perspective, symptoms such as anxiety, depression, interpersonal sensitivity, hostility, and maladaptation may not occur independently. Instead, they may mutually reinforce one another through direct pathways. For instance, interpersonal problems may increase emotional distress (Iovoli et al., 2025), anxiety may heighten academic pressure and maladaptation (Drăghici & Cazan, 2022), and depressive symptoms may reduce motivation and social engagement, thereby further aggravating interpersonal and school-related difficulties (Yang & Zhang, 2026). Thus, the ten symptom domains assessed in the present study can be understood as interrelated components of a dynamic psychological system. However, cross-sectional network analysis can capture only contemporaneous associations among variables and cannot reveal causal directionality or temporal dynamics. Given that adolescent mental health symptoms evolve over time, longitudinal research designs and methods are needed to clarify these complex dynamic relationships.

Compared with traditional network analysis, the recently developed Cross-Lagged Panel Network (CLPN) model can estimate temporal predictive effects among variables while simultaneously modeling the network structure among them, thereby revealing longitudinal processes through which symptom networks change both within and across time (Wysocki et al., 2025). In addition, by calculating out-expected influence (out-EI) and in-expected influence (in-EI), the CLPN model can identify which symptoms are most important in prospectively predicting other symptoms and which are most strongly predicted by other symptoms (Funkhouser et al., 2021; Wysocki et al., 2025). Symptoms with high out-EI may drive changes in other symptoms and may therefore be regarded as priority targets for intervention. Accordingly, the CLPN model is suitable for examining whether certain adolescent mental health dimensions show relatively stronger prospective associations with other symptom domains over time.

Given that the CLPN model requires multiple symptom domains as network nodes to examine temporal associations among symptoms, the present study employed the Middle School Student Mental Health Scale (MSSMHS). This scale provides a multidimensional assessment of adolescents’ psychological symptoms across ten domains: obsessive–compulsive symptoms, paranoid ideation, hostility, interpersonal sensitivity, depression, anxiety, academic stress, maladaptation, emotional instability, and psychological imbalance (J. Wang et al., 1997). These domains cover both internalizing symptoms, such as depression and anxiety, and interpersonal or school-related difficulties, such as interpersonal sensitivity, academic stress, and maladaptation, making the MSSMHS suitable for testing the network theory assumption that adolescent mental health problems are maintained through dynamic interactions among multiple symptom domains. Based on the network theory of psychopathology, the present study employed the CLPN model to examine prospective associations among broad adolescent mental health dimensions, with the aim of identifying central symptom domains and clarifying cross-temporal patterns of association among these domains. In addition, given that adolescent mental health problems and their network organization may vary across gender and developmental stages, this study further examined whether the temporal network structure differed by gender and grade level. By revealing longitudinal associations among symptom domains, this study may contribute to a more nuanced understanding of adolescent mental health and provide preliminary evidence for gender- and grade-sensitive screening, monitoring, and future intervention research.

## 2. Methods

### 2.1. Participants

This study used cluster sampling to recruit adolescents from a secondary school in Guangdong Province, China, and conducted a two-wave longitudinal survey via an online platform. The first wave of data collection was conducted between September and October 2023, and the second wave was conducted between March and April 2024, with an interval of approximately six months between assessments. Prior to data collection, informed consent was obtained from the participating school, participants’ guardians, and the participants themselves. Participants had the right to choose whether to participate and could withdraw at any time, and strict confidentiality was maintained throughout the study. A total of 3751 participants completed the survey at T1, and 3657 completed the survey at T2. After questionnaires with inattentive responses or excessively short completion times were excluded, data from the two waves were matched using student IDs. The final analytic sample consisted of 3610 participants aged 12 to 19 years (M = 15.26, SD = 1.66), including 1863 boys (51.6%) and 1747 girls (48.4%), as well as 1083 junior high school students (30.0%) and 2527 senior high school students (70.0%).

### 2.2. Measurements

#### Middle School Student Mental Health Scale (MSSMHS)

The Middle School Student Mental Health Scale (MSSMHS) was used to assess adolescents’ mental health problems (Dong & Dou, 2023; J. Wang et al., 1997). The scale consists of 60 items across 10 dimensions and is rated on a 5-point scale, with lower scores indicating fewer mental health problems and higher scores indicating more severe mental health problems. In the present study, Cronbach’s *α* coefficients for the total scale at T1 and T2 were 0.980 and 0.981, respectively. The subscales also demonstrated good reliability, with Cronbach’s *α* coefficients of 0.740 and 0.765 for obsessive–compulsive symptoms, 0.880 and 0.891 for paranoid ideation, 0.875 and 0.879 for hostility, 0.844 and 0.854 for interpersonal sensitivity, 0.883 and 0.888 for depression, 0.926 and 0.928 for anxiety, 0.879 and 0.882 for academic stress, 0.824 and 0.827 for maladaptation, 0.850 and 0.851 for emotional instability, and 0.839 and 0.855 for psychological imbalance at T1 and T2, respectively.

### 2.3. Statistical Analysis

Data were analyzed using R (version 4.5.2) (R Core Team, 2025), and correlational patterns were visualized using the corrplot package. To further examine directional relationships among variables, Cross-Lagged Panel Network (CLPN) analysis was conducted (Wysocki et al., 2025). After controlling for autoregressive effects (i.e., the effect of each node on itself from T1 to T2) and covariates, including gender, grade, and age, the cross-lagged effects of each node at T1 on other nodes at T2 were estimated to construct a directed network. In the overall sample model, gender, grade, and age were entered as covariates. For the subgroup models, the grouping variable itself was not included as a covariate: the gender-specific models controlled for grade and age, whereas the grade-specific models controlled for gender and age. This specification avoided adjustment for the variable used to define the subgroup while retaining adjustment for the remaining demographic covariates. The CLPN analysis comprised four steps. First, the glmnet package was used to estimate autoregressive and cross-lagged coefficients, and the penalty parameter that minimized cross-validation error was selected using 10-fold cross-validation. The least absolute shrinkage and selection operator (LASSO) regularization method was applied to shrink small regression coefficients to zero, thereby reducing false-positive edges and improving network interpretability (Friedman et al., 2010). Second, the qgraph package was used to visualize the CLPN, providing a clear representation of the directional relationships among nodes (Epskamp et al., 2012, 2018). Third, the qgraph package was used to calculate centrality indices of the CLPN, including out-expected influence (out-EI) and in-expected influence (in-EI). Specifically, out-EI was defined as the sum of the weights of all outgoing edges from a given node, reflecting the extent to which that node predicted other nodes in the network. In contrast, in-EI was defined as the sum of the weights of all incoming edges to a given node, reflecting the extent to which that node was predicted by other nodes in the network (Funkhouser et al., 2021; Wysocki et al., 2025). Fourth, the bootnet package was used to assess the stability and accuracy of the CLPN via bootstrapping (Epskamp et al., 2018). The 95% confidence intervals of edge weights were estimated using a nonparametric bootstrap procedure with 1000 iterations to assess the accuracy of edge-weight estimation. In addition, the correlation stability coefficient (CS-coefficient) was calculated using a case-dropping bootstrap procedure with 1000 iterations to evaluate the stability of centrality indices. Specifically, a CS-coefficient greater than 0.25 was considered acceptable; values between 0.20 and 0.25 remained open to discussion, but conclusions should be drawn cautiously in light of other research findings; and values below 0.20 indicated insufficient stability and were therefore not suitable for further interpretation or discussion.

## 3. Results

### 3.1. Correlation Analysis

**Figure 1:**
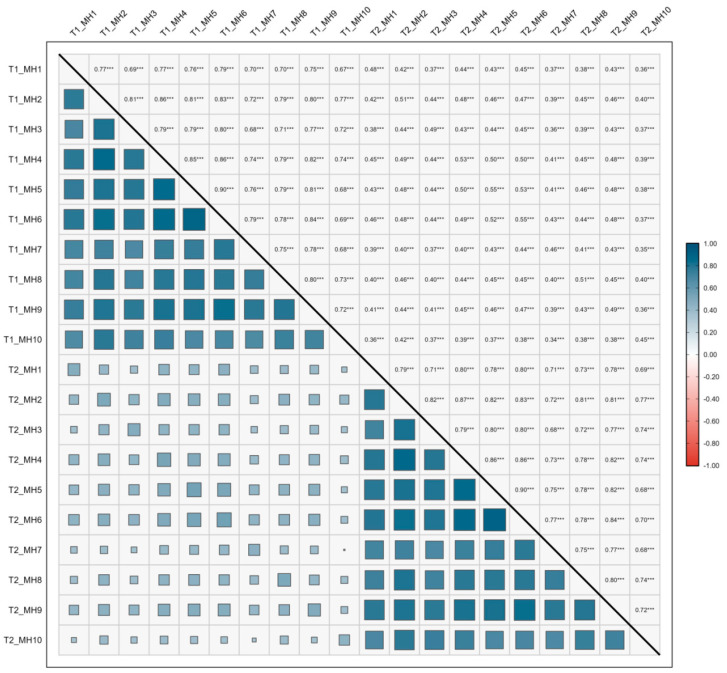
Correlations among variables of adolescent mental health. Note: *** *p* < 0.001. The same below.

The specific node names examined in the present study, along with their means and standard deviations, are presented in Table 1. Figure 1 shows the correlation heatmap of the variables measured at T1 and T2. Overall, the dimensions of adolescent mental health showed moderate to strong positive correlations across the two time points.

### 3.2. CLPN Results

Before interpreting the CLPN results, measurement invariance analyses showed that the MSSMHS achieved configural, metric, scalar, and strict invariance across time, gender, and grade level (see Supplementary Materials Table S1), indicating that the scale measured comparable constructs across waves and groups and supporting the interpretation of longitudinal and group-based network differences.

**Figure 2:**
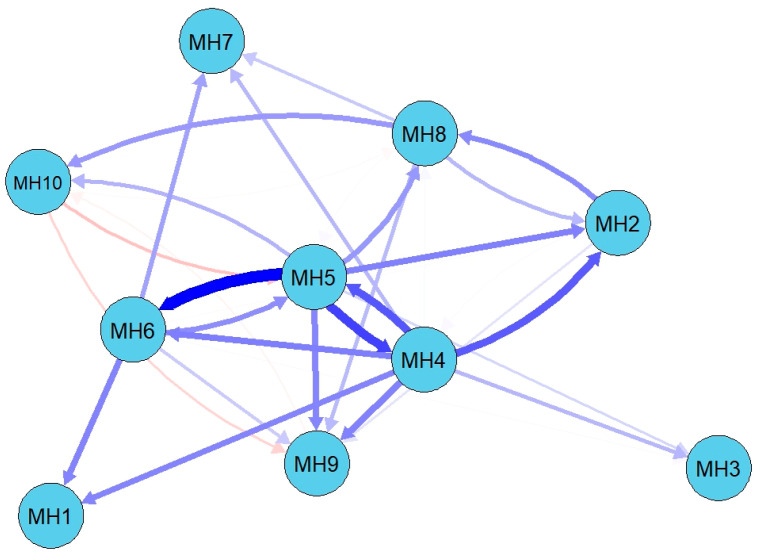
Total sample Cross-Lagged Panel Network. Note: Arrows indicate the direction of the cross-lagged relationships. Blue edges represent positive relationships, whereas red edges represent negative relationships. Edge thickness corresponds to the magnitude of the edge weights, with thicker edges indicating stronger relationships. The detailed descriptions and abbreviations of all nodes are provided in the legend. To facilitate visual interpretation, autoregressive edges, weaker edges with absolute edge weights smaller than 0.03, and covariates were excluded from the diagram.

To improve the clarity of the cross-lagged paths among variables, edges with absolute values smaller than 0.03 were hidden in the network graph. This threshold was used solely for graphical presentation and did not constitute a substantive criterion for interpreting, retaining, or excluding effects. Therefore, the interpretation of the network was based on the estimated edge weights, 95% confidence intervals, and centrality indices rather than on the 0.03 plotting threshold. The Cross-Lagged Panel Network with very small edges visually suppressed is shown in Figure 2. The plotting algorithm scaled the thickness of the paths according to their strength to enhance interpretability. Blue arrows indicate positive predictive relationships, whereas red arrows indicate negative predictive relationships. In the T1→T2 cross-lagged network, interpersonal sensitivity (MH4) showed the broadest outgoing predictive influence, predicting depression (MH5), paranoid ideation (MH2), obsessive–compulsive symptoms (MH1), and emotional instability (MH9). The three strongest cross-lagged edges in the network were as follows: depression (MH5) positively predicted anxiety (MH6; edge weight = 0.15, 95% CI = [0.07, 0.24]); depression (MH5) positively predicted interpersonal sensitivity (MH4; edge weight = 0.12, 95% CI = [0.05, 0.19]); and interpersonal sensitivity (MH4) positively predicted depression (MH5; edge weight = 0.11, 95% CI = [0.03, 0.19]).

**Figure 3:**
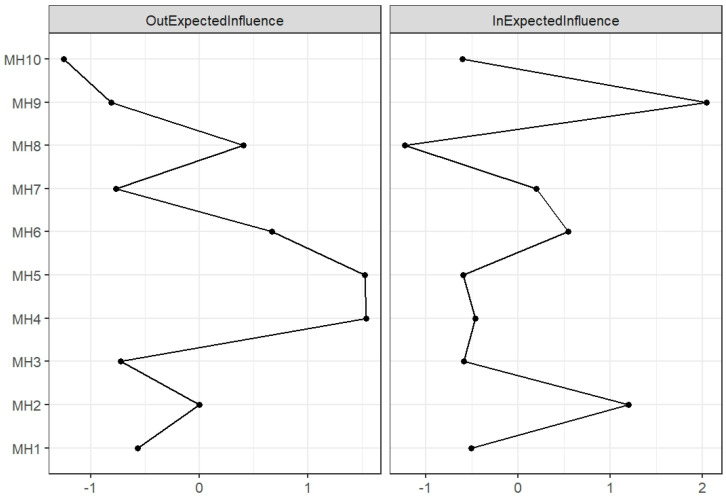
Estimated centrality indices values for the total sample Cross-Lagged Panel Network (out-EI and in-EI).

The centrality estimates of the nodes are presented in Figure 3. Interpersonal sensitivity (MH4) had the highest out-EI (out-EI = 0.68), indicating that it exerted the strongest predictive influence on other factors in the network, followed by depression (MH5; out-EI = 0.67) and anxiety (MH6; out-EI = 0.42). Emotional instability (MH9) had the highest in-EI (in-EI = 0.32), suggesting that it was most strongly predicted by other factors in the network, followed by paranoid ideation (MH2; in-EI = 0.28) and anxiety (MH6; in-EI = 0.24). The bootstrap analysis indicated acceptable precision of the edge-weight estimates, as the confidence interval widths remained within a reasonable range. In addition, in the T1→T2 network, the correlation stability coefficients for out-EI and in-EI were both 0.44 (for details, see Supplementary Figures S1–S5).

### 3.3. Gender Differences

To examine gender differences across variables, independent-samples t tests were conducted for the variables at T1 and T2. The results (see Table 2) showed that, at both T1 and T2, female students scored significantly higher than male students on nine variables: obsessive–compulsive symptoms, paranoid ideation, hostility, interpersonal sensitivity, depression, anxiety, academic stress, maladaptation, and emotional instability (*p* < 0.001). Only psychological imbalance showed no significant gender difference (*p* > 0.05). Given the large sample size, Cohen’s *d* was further calculated to evaluate the practical magnitude of these gender differences. The effect sizes for significant gender differences were generally small, ranging from *d* = 0.17 to 0.38 at T1 and from *d* = 0.11 to 0.33 at T2. The largest gender differences were observed for depression, anxiety, and academic stress, with female students reporting higher scores than male students; however, these effects remained in the small to small-to-moderate range. The effect size for psychological imbalance was negligible at both T1 (*d* = 0.06) and T2 (*d* = 0.02), consistent with the nonsignificant group differences. Therefore, using the ten variables as nodes, cross-lagged network models were constructed separately for male and female students to examine gender-specific patterns of cross-temporal associations among mental health domains.

**Figure 4:**
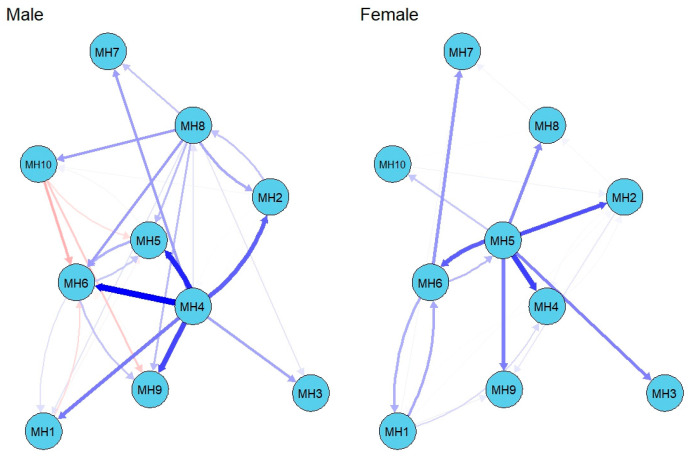
Male and female Cross-Lagged Panel Networks. Note: Arrows indicate the direction of the cross-lagged relationships. Blue edges represent positive relationships, whereas red edges represent negative relationships. Edge thickness corresponds to the magnitude of the edge weights, with thicker edges indicating stronger relationships. The detailed descriptions and abbreviations of all nodes are provided in the legend. To facilitate visual interpretation, autoregressive edges, weaker edges with absolute edge weights smaller than 0.03, and covariates were excluded from the diagram.

**Figure 5:**
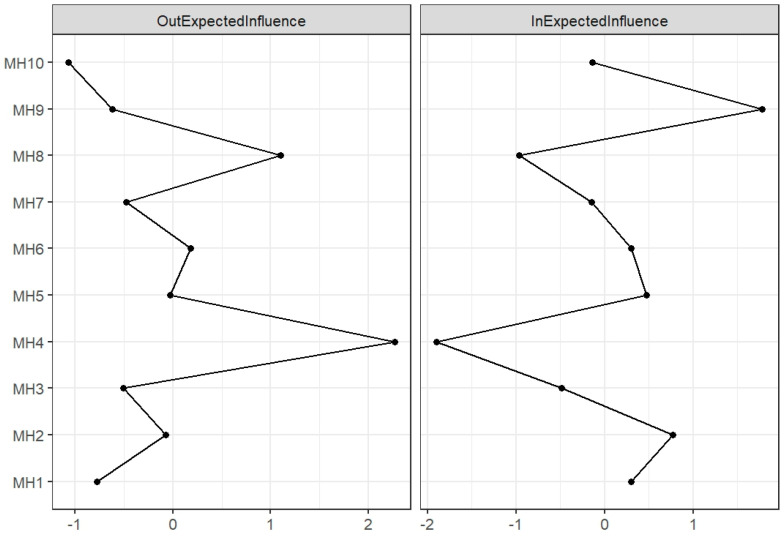
Estimated centrality indices values for the male Cross-Lagged Panel Network (out-EI and in-EI).

In the male network, interpersonal sensitivity (MH4) showed the most extensive outgoing predictive effects, predicting anxiety (MH6), depression (MH5), emotional instability (MH9), and paranoid ideation (MH2). Among male students, the most prominent cross-lagged paths were concentrated around interpersonal sensitivity: interpersonal sensitivity (MH4) positively predicted anxiety (MH6; edge weight = 0.22, 95% CI = [0.09, 0.33]), depression (MH5; edge weight = 0.20, 95% CI = [0.10, 0.30]), and emotional instability (MH9; edge weight = 0.17, 95% CI = [0.07, 0.27]) (see Figure 4). The node centrality estimates are presented in Figure 5. Interpersonal sensitivity (MH4) showed the highest out-EI (out-EI = 1.16), followed by maladaptation (MH8; out-EI = 0.68) and anxiety (MH6; out-EI = 0.30). Emotional instability (MH9) showed the highest in-EI (in-EI = 0.36), followed by paranoid ideation (MH2; in-EI = 0.28) and depression (MH5; in-EI = 0.26). Bootstrap results supported the accuracy of the edge-weight estimates, with confidence interval widths falling within an acceptable range. In addition, in the male network, the correlation stability coefficients for out-EI and in-EI were both 0.44 (for details, see Supplementary Figures S6–S10).

**Figure 6:**
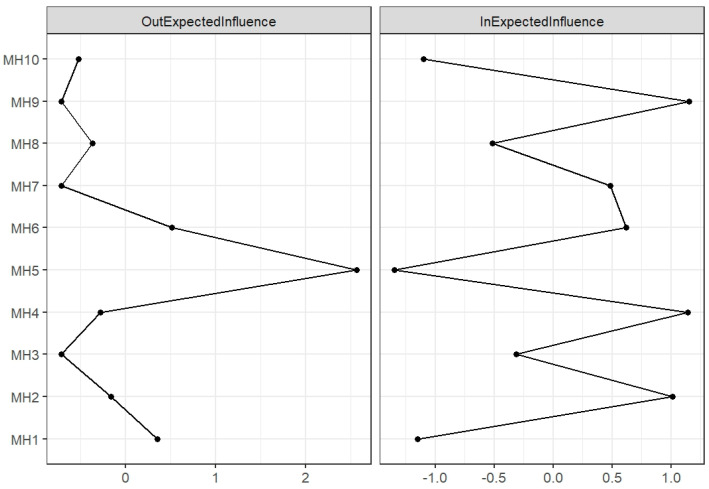
Estimated centrality indices values for the female Cross-Lagged Panel Network (out-EI and in-EI).

In the female network, depression (MH5) displayed the strongest outgoing predictive role, predicting interpersonal sensitivity (MH4), paranoid ideation (MH2), anxiety (MH6), and emotional instability (MH9). In contrast to the male network, the strongest predictive effects in the female network mainly originated from depression: depression (MH5) positively predicted interpersonal sensitivity (MH4; edge weight = 0.18, 95% CI = [0.09, 0.27]), paranoid ideation (MH2; edge weight = 0.16, 95% CI = [0.08, 0.25]), and anxiety (MH6; edge weight = 0.16, 95% CI = [0.05, 0.27]) (see Figure 4). The node centrality estimates are shown in Figure 6. Depression (MH5) had the highest out-EI (out-EI = 0.97), followed by anxiety (MH6; out-EI = 0.36) and obsessive–compulsive symptoms (MH1; out-EI = 0.32). Emotional instability (MH9) had the highest in-EI (in-EI = 0.29), followed by interpersonal sensitivity (MH4; in-EI = 0.29) and paranoid ideation (MH2; in-EI = 0.28). The edge-weight bootstrap analysis again suggested adequate estimation accuracy, as the confidence interval widths were within an acceptable range. In addition, in the female network, the correlation stability coefficients for out-EI and in-EI were both 0.28 (for details, see Supplementary Figures S11–S15).

### 3.4. Grade Differences

To examine grade differences across variables, independent-samples *t* tests were conducted separately for the variables measured at T1 and T2. The results (see Table 3) showed that, except for academic stress at T2, senior high school students scored significantly higher than junior high school students on all other variables (*p* < 0.001). To avoid overinterpreting statistically significant differences in the context of a large sample, Cohen’s *d* was also calculated for each grade comparison. The effect sizes indicated that most grade differences were small to small-to-moderate in magnitude. At T1, Cohen’s *d* values ranged from 0.17 to 0.50, with the largest difference observed for maladaptation, followed by depression, interpersonal sensitivity, and psychological imbalance. At T2, the effect sizes were generally smaller, ranging from 0.14 to 0.31. The difference in academic stress at T2 was negligible (*d* = 0.04), consistent with the nonsignificant *t*-test result. Overall, these findings suggest that although senior high school students tended to report higher levels of most mental health symptoms than junior high school students, the practical magnitude of these differences was generally limited. Therefore, using the 10 variables as nodes, cross-lagged network models were constructed separately for junior and senior high school students to examine grade-specific patterns of cross-temporal associations among mental health domains.

**Figure 7:**
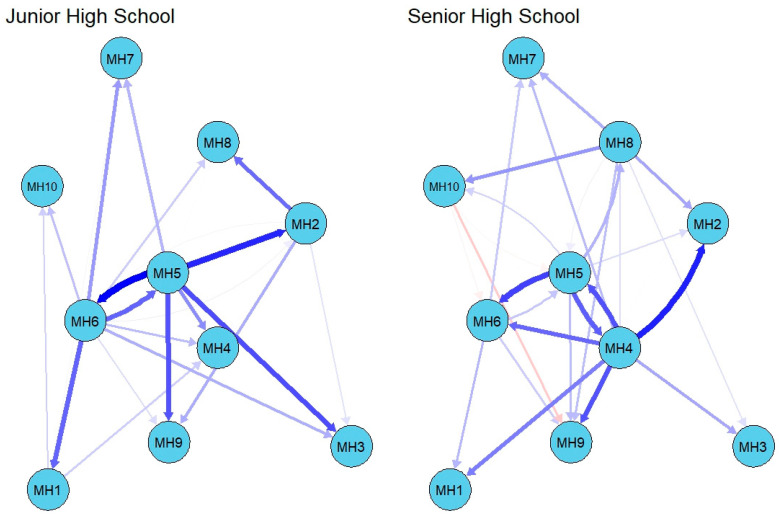
Junior high school and senior high school Cross-Lagged Panel Networks. Note: Arrows indicate the direction of the cross-lagged relationships. Blue edges represent positive relationships, whereas red edges represent negative relationships. Edge thickness corresponds to the magnitude of the edge weights, with thicker edges indicating stronger relationships. The detailed descriptions and abbreviations of all nodes are provided in the legend. To facilitate visual interpretation, autoregressive edges, weaker edges with absolute edge weights smaller than 0.03, and covariates were excluded from the diagram.

**Figure 8:**
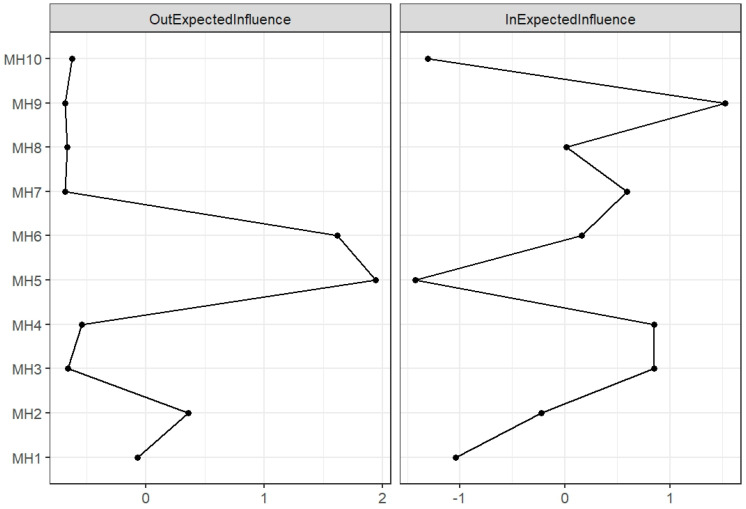
Estimated centrality indices values for the junior high school Cross-Lagged Panel Network (out-EI and in-EI).

In the junior high school network, depression (MH5) showed the widest range of outgoing predictive effects, predicting anxiety (MH6), paranoid ideation (MH2), hostility (MH3), and emotional instability (MH9). The strongest cross-lagged associations in this network were primarily driven by depression: depression (MH5) positively predicted anxiety (MH6; edge weight = 0.18, 95% CI = [0.02, 0.34]), paranoid ideation (MH2; edge weight = 0.16, 95% CI = [0.02, 0.29]), and hostility (MH3; edge weight = 0.14, 95% CI = [0.01, 0.26]) (see Figure 7). The node centrality estimates are presented in Figure 8. Depression (MH5) showed the highest out-EI (out-EI = 0.80), followed by anxiety (MH6; out-EI = 0.70) and paranoid ideation (MH2; out-EI = 0.32). Emotional instability (MH9) showed the highest in-EI (in-EI = 0.30), followed by interpersonal sensitivity (MH4; in-EI = 0.26) and hostility (MH3; in-EI = 0.26). The bootstrap procedure indicated acceptable accuracy of the edge-weight estimates. In addition, in the junior high school network, the correlation stability coefficients for out-EI and in-EI were both 0.28 (for details, see Supplementary Figures S16–S20).

**Figure 9:**
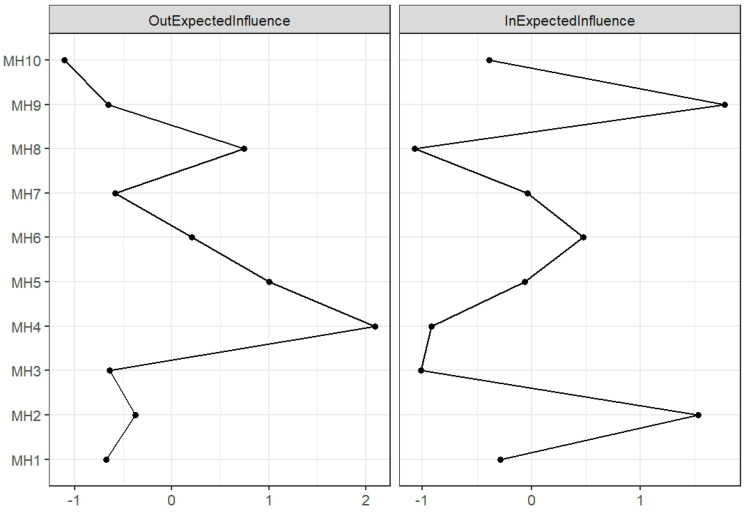
Estimated centrality indices values for the senior high school Cross-Lagged Panel Network (out-EI and in-EI).

In the senior high school network, interpersonal sensitivity (MH4) emerged as the node with the greatest number of outgoing predictive effects, predicting paranoid ideation (MH2), anxiety (MH6), emotional instability (MH9), and depression (MH5), among others. The most pronounced predictive paths in this network were as follows: interpersonal sensitivity (MH4) positively predicted paranoid ideation (MH2; edge weight = 0.15, 95% CI = [0.07, 0.25]); depression (MH5) positively predicted anxiety (MH6; edge weight = 0.13, 95% CI = [0.04, 0.24]); and interpersonal sensitivity (MH4) positively predicted emotional instability (MH9; edge weight = 0.13, 95% CI = [0.04, 0.22]) (see Figure 7). Node centrality estimates (see Figure 9) indicated that interpersonal sensitivity (MH4) had the highest out-EI (out-EI = 0.88), followed by depression (MH5; out-EI = 0.54) and maladaptation (MH8; out-EI = 0.45). Emotional instability (MH9) had the highest in-EI (in-EI = 0.32), followed by paranoid ideation (MH2; in-EI = 0.31) and anxiety (MH6; in-EI = 0.24). The edge-weight bootstrap results showed adequate estimation accuracy, with confidence interval widths remaining within an acceptable range. In addition, in the senior high school network, the correlation stability coefficients for out-EI and in-EI were both 0.36 (for details, see Supplementary Figures S21–S25).

## 4. Discussion

### 4.1. Central Symptom Domains in Adolescent Mental Health

First, in the cross-lagged network of the overall sample, interpersonal sensitivity (MH4) exhibited the highest out-EI value, followed by depression (MH5) and anxiety (MH6). This indicates that interpersonal sensitivity showed the strongest outgoing prospective associations with subsequent mental health dimensions within the adolescent mental health symptom network. Moreover, interpersonal sensitivity not only showed multiple cross-temporal associations with other domains but also showed a strong bidirectional association with depression. Specifically, depression significantly predicted subsequent interpersonal sensitivity, while interpersonal sensitivity, in turn, significantly predicted subsequent depression. These findings suggest a close reciprocal prospective linkage between interpersonal sensitivity and depression in adolescent mental health development (Cohen et al., 2015; Li et al., 2020). According to interpersonal theory, adolescents are highly sensitive to peer feedback during this developmental stage; interpersonal conflict, rejection experiences, and relational strain may contribute to negative self-representations, thereby contributing to internalizing symptoms such as depression and anxiety (Pagliaccio et al., 2023). Conversely, depressive states characterized by withdrawal, negative attributional styles, and low perceived social efficacy may impair interpersonal functioning, increasing the likelihood of tension, misunderstanding, and social disconnection in peer interactions (Hammen, 1991, 2012; McLaughlin & Nolen-Hoeksema, 2012). Thus, the bidirectional edge between interpersonal sensitivity and depression highlights the potential importance of the interpersonal–depressive interface in understanding adolescent mental health problems.

Second, depression also demonstrated a relatively high out-EI value in the overall network and positively predicted both anxiety and interpersonal sensitivity. Cognitive theories suggest that individuals with depression often exhibit negative cognitive biases, low self-worth, and pessimistic expectations about the future, which may amplify threat perception and uncertainty and thereby elicit anxiety (Abramson et al., 1989; Hankin et al., 2004). This pattern is consistent with the present finding that depression was prospectively associated with subsequent anxiety and interpersonal sensitivity in the overall network. Together, these results suggest that depressive symptoms may play an important role in the temporal organization of adolescent internalizing and interpersonal difficulties.

Finally, in the overall network, emotional instability showed the highest in-EI value, followed by paranoid ideation and anxiety. This suggests that emotional instability was the domain most strongly associated with incoming cross-temporal prediction from other mental health dimensions. In other words, emotional instability may be better understood as a sensitive indicator of broader psychological difficulties rather than as the primary initiating node. Emotion regulation theory posits that, during adolescence, an imbalance between neurobiological maturation and social contextual demands renders the emotion regulation system relatively fragile (Cristofanelli et al., 2024). When domains such as depression, anxiety, and interpersonal sensitivity accumulate to a certain extent, they are more likely to manifest as emotional instability.

### 4.2. Gender Differences in Adolescent Mental Health Domains

The present study found that, at both time points, girls scored significantly higher than boys on nine symptom domains, with the exception of psychological imbalance. This finding is consistent with prior research indicating higher levels of internalizing problems among adolescent girls (Hankin, 2009; Nolen-Hoeksema & Girgus, 1994). However, effect-size analyses showed that these gender differences were generally small, with Cohen’s *d* values ranging from 0.17 to 0.38 at T1 and from 0.11 to 0.33 at T2. Therefore, although girls reported higher symptom levels than boys, the practical magnitude of these differences should be interpreted cautiously. More importantly, the cross-lagged network analysis further suggested that boys and girls differed not only in symptom levels but also in the pattern of cross-temporal associations among mental health domains.

In the male network, interpersonal sensitivity exhibited the highest out-EI value, and the three strongest pathways all originated from interpersonal sensitivity, predicting anxiety, depression, and emotional instability. Gender role socialization theory suggests that boys are often expected to display independence, emotional restraint, and competitiveness, which may make them less likely to directly express vulnerability when facing interpersonal conflict or relational setbacks. Instead, such distress may be expressed indirectly through anxiety, depression, or emotional dysregulation (Zamarripa et al., 2003). During adolescence, when peer relationships become central to identity formation, threats to belonging, peer status, or relational evaluation may therefore be linked to broader emotional symptoms among boys.

In contrast, in the female network, depression exhibited the highest out-EI value, and the three strongest pathways all originated from depression, predicting interpersonal sensitivity, paranoid ideation, and anxiety. Previous studies have shown that girls tend to display greater emotional sensitivity and a stronger tendency toward rumination during adolescence, making them more likely to dwell on negative emotional experiences following stressors or interpersonal difficulties (Rose, 2002). Once depressive symptoms emerge, they may shape girls’ interpretations of others’ intentions through negative attributional styles, self-devaluation, and heightened interpersonal vigilance, thereby increasing interpersonal sensitivity and paranoid ideation (Lee, 2017). Depression may also intensify perceived uncertainty and threat, which could further contribute to anxiety (Cummings et al., 2014; Kim et al., 2020).

Taken together, these findings suggest that gender differences in adolescent mental health may be reflected not only in mean-level differences but also in the organization of prospective associations among mental health dimensions. For boys, interpersonal sensitivity may represent a candidate domain for screening and monitoring, whereas for girls, the depression domain may warrant particular attention in early identification and prevention efforts.

### 4.3. Grade Differences in Adolescent Mental Health Domains

The present study further found that, except for T2 academic stress, senior high school students scored significantly higher than junior high school students across all other mental health dimensions, indicating an overall increase in mental health risk with advancing grade level. However, the effect-size results showed that most grade differences were small to small-to-moderate in magnitude. At T1, Cohen’s *d* values ranged from 0.17 to 0.50, whereas at T2 they ranged from 0.14 to 0.31, with the difference in academic stress at T2 being negligible. Therefore, although senior high school students tended to report higher levels across several mental health dimensions, these mean-level differences should not be overinterpreted. This pattern is consistent with the increased academic demands, more complex social evaluation, and intensified identity formation pressures encountered during late adolescence (S. Wang et al., 2025).

However, different grade groups also exhibited distinct patterns in cross-temporal symptom associations. In the junior high school network, depression exhibited the highest out-EI value, followed by anxiety and paranoid ideation, with the strongest pathways showing depression predicting anxiety, paranoid ideation, and hostility. This suggests that, among junior high school students, depression showed relatively stronger outgoing prospective associations with subsequent emotional, cognitive, and behavioral difficulties. Junior high school corresponds to early-to-middle adolescence, a period during which emotional experiences, self-concept, and social cognition remain relatively unstable, making internal emotional distress more likely to serve as a starting point for psychological problems (Patel et al., 2007). At this stage, depression may not only be associated with anxiety and hostility but also influence how individuals interpret others’ evaluations and social information, thereby increasing paranoid experiences (Januška et al., 2021; S. Wang et al., 2025).

In contrast, in the senior high school network, interpersonal sensitivity exhibited the highest out-EI value, followed by depression and maladaptation. The strongest pathways indicated that interpersonal sensitivity predicted paranoid ideation and emotional instability, whereas depression predicted anxiety. As adolescents enter late adolescence, they are confronted with more complex peer networks, more salient social comparisons, and stronger academic competition pressures (Jayanthi et al., 2015; Tang et al., 2019). Consequently, psychological distress among senior high school students may be more closely embedded in interpersonal and environmental adaptation contexts. Under such conditions, interpersonal sensitivity may influence adolescents’ sense of belonging, social-evaluative experiences, and interpersonal security, thereby contributing to broader difficulties across multiple mental health domains, including paranoid ideation, anxiety, depression, and emotional instability (Cohen et al., 2015).

Overall, the grade-specific network findings suggest that screening and monitoring priorities may differ across developmental stages. For junior high school students, the depression domain may warrant particular attention, as depression showed relatively stronger outgoing predictive associations with other domains. For senior high school students, interpersonal sensitivity and adaptation to social–academic stressors may represent important areas for monitoring and future intervention research.

## 5. Practical Implications

Based on the CLPN model, this study identified mental health dimensions with relatively strong cross-temporal predictive associations, as well as dimensions that were more strongly predicted by other domains. These findings suggest that adolescent mental health dimensions are prospectively interrelated rather than statistically independent. Among these domains, interpersonal sensitivity and depression demonstrated relatively strong outgoing predictive associations and may help identify priority areas for screening, early warning, monitoring, and future intervention research in school mental health services. In contrast, emotional instability, paranoid ideation, and anxiety showed stronger incoming associations and may serve as important indicators for monitoring changes in adolescents’ mental health status.

Furthermore, the study suggested that the relative prominence of predictive domains varied across gender and grade groups. Interpersonal sensitivity showed relatively stronger outgoing associations among boys, whereas depression showed relatively stronger outgoing associations among girls. Similarly, among junior high school students, depression showed relatively stronger outgoing predictive associations, whereas among senior high school students, interpersonal sensitivity showed relatively stronger outgoing predictive associations. Therefore, school-based mental health programs should avoid uniform, one-size-fits-all approaches and may benefit from incorporating gender- and grade-sensitive screening and monitoring strategies.

## 6. Limitations and Future Directions

Despite using the CLPN approach to examine cross-temporal associations among adolescent mental health symptoms and further exploring gender and grade differences, this study has several limitations.

First, the study relied primarily on self-report questionnaire data, which may be subject to social desirability bias, common method bias, and individual differences in subjective interpretation. When reporting their own mental health status, adolescents may provide biased responses due to variations in self-awareness, current emotional state, or concerns about disclosing sensitive information. In addition, in the present network analysis, the ten dimensions of the Middle School Student Mental Health Scale (MSSMHS) were used as nodes. Therefore, the resulting network reflects associations among broad mental health dimensions rather than associations among individual symptom items. Although dimension-level nodes are useful for summarizing broader domains of adolescent mental health, they may obscure more fine-grained symptom-to-symptom associations. Thus, the present findings should be interpreted as a domain-level prospective network rather than as an item-level symptom network. Future research should incorporate multiple data sources, such as peer nominations, parent reports, teacher ratings, and clinical interviews, and further examine item-level symptom networks to enhance the robustness, precision, and ecological validity of the findings.

Second, the present study included only two measurement waves. Although this design allowed for a preliminary examination of cross-temporal predictive relationships among mental health symptoms, it was insufficient to fully capture continuous developmental changes in symptom networks over time. It also did not allow for a finer-grained examination of potential nonlinear changes or stage-specific transitions among symptoms. Future studies should adopt longer-term, multi-wave longitudinal designs to more accurately characterize the developmental trajectories and dynamic mechanisms of adolescent mental health symptoms.

Third, although the subgroup analyses provided useful information about gender- and grade-specific network patterns, these analyses were based on separately estimated networks. Therefore, the observed differences in subgroup network patterns should be interpreted cautiously and should not be taken as formal statistical evidence of between-group differences in network structure, edge weights, or centrality indices. Future research could use formal network comparison approaches or moderated network models to examine whether gender- and grade-related differences in prospective associations are statistically robust.

Fourth, the sample was drawn exclusively from middle school students in Guangdong Province, which may limit its representativeness. Given potential regional differences in socioeconomic background, educational context, and school-based mental health resources, caution is warranted when generalizing the present findings to adolescents from other regions of China or from different educational settings. Future research should expand the sampling scope by including adolescents from different provinces and various types of schools, thereby improving the external validity and generalizability of the findings.

## 7. Conclusions

This study examined prospective longitudinal associations among adolescent mental health dimensions using a two-wave CLPN model. In the overall network, interpersonal sensitivity and depression showed relatively stronger outgoing predictive associations, whereas emotional instability was the dimension most strongly predicted by other domains. In the subgroup analyses, interpersonal sensitivity showed the highest out-EI in the male network, whereas depression showed the highest out-EI in the female network. Depression showed the highest out-EI in the junior high school network, whereas interpersonal sensitivity showed the highest out-EI in the senior high school network. These findings suggest that the organization of adolescent mental health dimensions may vary by gender and grade level. Future school-based mental health services may consider these gender- and grade-specific patterns when developing screening, monitoring, and prevention strategies.

## Figures and Tables

**Table 1:** Item labels and descriptive statistics at two time points (n = 3610).

Variable	Label	T1		T2	
		*M*	*SD*	*M*	*SD*
Obsessive–compulsive symptoms	MH1	1.99	0.66	2.01	0.68
Paranoid ideation	MH2	1.74	0.71	1.82	0.74
Hostility	MH3	1.60	0.69	1.67	0.72
Interpersonal sensitivity	MH4	1.84	0.74	1.90	0.76
Depression	MH5	1.87	0.80	1.93	0.82
Anxiety	MH6	1.94	0.90	2.00	0.90
Academic stress	MH7	2.03	0.86	2.07	0.87
Maladaptation	MH8	1.83	0.69	1.90	0.71
Emotional instability	MH9	2.00	0.75	2.07	0.77
Psychological imbalance	MH10	1.54	0.61	1.60	0.64

**Table 2:** Gender differences in all variables (n = 3610).

Variable	T1				T2			
	Male (n = 1863)	Female (n = 1747)	*t*	Cohen’s *d*	Male (n = 1863)	Female (n = 1747)	*t*	Cohen’s *d*
Obsessive–compulsive symptoms	1.93 ± 0.65	2.06 ± 0.67	−6.12 ***	0.20	1.95 ± 0.68	2.07 ± 0.67	−5.49 ***	0.18
Paranoid ideation	1.68 ± 0.69	1.81 ± 0.72	−5.29 ***	0.18	1.77 ± 0.74	1.87 ± 0.74	−4.16 ***	0.14
Hostility	1.54 ± 0.66	1.66 ± 0.72	−5.20 ***	0.17	1.63 ± 0.73	1.71 ± 0.72	−3.38 **	0.11
Interpersonal sensitivity	1.76 ± 0.74	1.92 ± 0.74	−6.20 ***	0.21	1.83 ± 0.76	1.97 ± 0.76	−5.30 ***	0.18
Depression	1.72 ± 0.73	2.02 ± 0.84	−11.40 ***	0.38	1.81 ± 0.77	2.07 ± 0.85	−9.81 ***	0.33
Anxiety	1.80 ± 0.85	2.08 ± 0.93	−9.44 ***	0.32	1.87 ± 0.86	2.12 ± 0.93	−8.34 ***	0.28
Academic stress	1.92 ± 0.85	2.15 ± 0.86	−8.09 ***	0.27	1.96 ± 0.86	2.18 ± 0.86	−7.91 ***	0.26
Maladaptation	1.77 ± 0.69	1.89 ± 0.69	−5.36 ***	0.18	1.85 ± 0.73	1.96 ± 0.69	−4.52 ***	0.15
Emotional instability	1.92 ± 0.75	2.08 ± 0.75	−6.48 ***	0.22	2.00 ± 0.76	2.15 ± 0.77	−6.16 ***	0.21
Psychological imbalance	1.52 ± 0.62	1.56 ± 0.60	−1.73	0.06	1.59 ± 0.66	1.60 ± 0.63	−0.47	0.02

** *p* < 0.01, *** *p* < 0.001.

**Table 3:** Grade differences in all variables (n = 3610).

Variable	T1				T2			
	Junior High School (n = 1083)	Senior High School (n = 2527)	*t*	Cohen’s *d*	Junior High School (n = 1083)	Senior High School (n = 2527)	*t*	Cohen’s *d*
Obsessive–compulsive symptoms	1.89 ± 0.63	2.04 ± 0.67	−6.53 ***	0.23	1.94 ± 0.67	2.04 ± 0.68	−3.83 ***	0.14
Paranoid ideation	1.59 ± 0.65	1.81 ± 0.72	−8.72 ***	0.30	1.70 ± 0.72	1.87 ± 0.75	−6.47 ***	0.23
Hostility	1.52 ± 0.67	1.64 ± 0.70	−4.88 ***	0.17	1.60 ± 0.72	1.70 ± 0.73	−3.51 ***	0.13
Interpersonal sensitivity	1.66 ± 0.68	1.91 ± 0.75	−9.93 ***	0.35	1.78 ± 0.74	1.94 ± 0.77	−5.86 ***	0.21
Depression	1.67 ± 0.76	1.96 ± 0.80	−10.19 ***	0.36	1.80 ± 0.81	1.99 ± 0.82	−6.52 ***	0.24
Anxiety	1.74 ± 0.88	2.02 ± 0.90	−8.63 ***	0.31	1.87 ± 0.90	2.05 ± 0.90	−5.62 ***	0.20
Academic stress	1.88 ± 0.86	2.09 ± 0.85	−6.89 ***	0.25	2.05 ± 0.90	2.08 ± 0.85	−0.98	0.04
Maladaptation	1.59 ± 0.60	1.93 ± 0.70	−14.58 ***	0.50	1.75 ± 0.67	1.97 ± 0.72	−8.76 ***	0.31
Emotional instability	1.84 ± 0.73	2.07 ± 0.75	−8.42 ***	0.31	1.99 ± 0.76	2.11 ± 0.77	−4.23 ***	0.15
Psychological imbalance	1.40 ± 0.48	1.60 ± 0.65	−10.14 ***	0.33	1.50 ± 0.58	1.64 ± 0.67	−6.05 ***	0.21

*** *p* < 0.001.

## Data Availability

The data that support the findings of this study are available upon request from the corresponding author.

## Supplementary Materials

The following supporting information can be downloaded at https://www.mdpi.com/article/10.3390/bs16060928/s1.

## Abbreviations

The following abbreviations are used in this manuscript:

MH1	obsessive–compulsive symptoms
MH2	paranoid ideation
MH3	hostility
MH4	interpersonal sensitivity
MH5	depression
MH6	anxiety
MH7	academic stress
MH8	maladaptation
MH9	emotional instability
MH10	psychological imbalance
CS-coefficient	correlation stability coefficient
out-EI	out-expected influence
in-EI	in-expected influence
CLPN	Cross-Lagged Panel Network
