# Supplementary material for: Dynamic Network Characteristics of Adolescent Mental Health Symptoms: Gender and Grade Differences Based on a Cross-Lagged Panel Network Model
Source: Behav Sci (Basel). 2026 Jun 5;16(6):928. doi: 10.3390/bs16060928 (PMC13295604; doi:10.3390/bs16060928)
Supplement: Supplementary file 1 [file behavsci-16-00928-s001.zip › behavsci-4296132-supplementary.pdf]

Table S1. Measurement invariance of the MSSMHS across time, gender, and grade level.

Invariance Test	Model	CFI	TLI	RMSEA	SRMR	Δ CFI	Δ RMSEA	Δ SRM
T1 vs. T2	Configural	0.965	0.959	0.075	0.021	-	-	-
T1 vs. T2	Metric	0.965	0.961	0.073	0.028	0.000	-0.002	0.007
T1 vs. T2	Scalar	0.964	0.962	0.071	0.028	-0.001	-0.001	0.000
T1 vs. T2	Strict	0.964	0.964	0.070	0.029	0.000	-0.002	0.000
Gender	Configural	0.967	0.960	0.073	0.020	-	-	-
Gender	Metric	0.965	0.960	0.073	0.034	-0.002	0.000	0.014
Gender	Scalar	0.961	0.958	0.075	0.036	-0.004	0.002	0.002
Gender	Strict	0.960	0.959	0.074	0.037	-0.001	-0.001	0.001
Grade	Configural	0.965	0.958	0.075	0.022	-	-	-
Grade	Metric	0.963	0.958	0.075	0.037	-0.002	0.000	0.014
Grade	Scalar	0.960	0.957	0.076	0.037	-0.003	0.001	0.001
Grade	Strict	0.958	0.958	0.075	0.040	-0.002	0.000	0.003

Note. All values were rounded to three decimal places. Accordingly, values with absolute magnitudes smaller than 0.001 are displayed as 0.000.

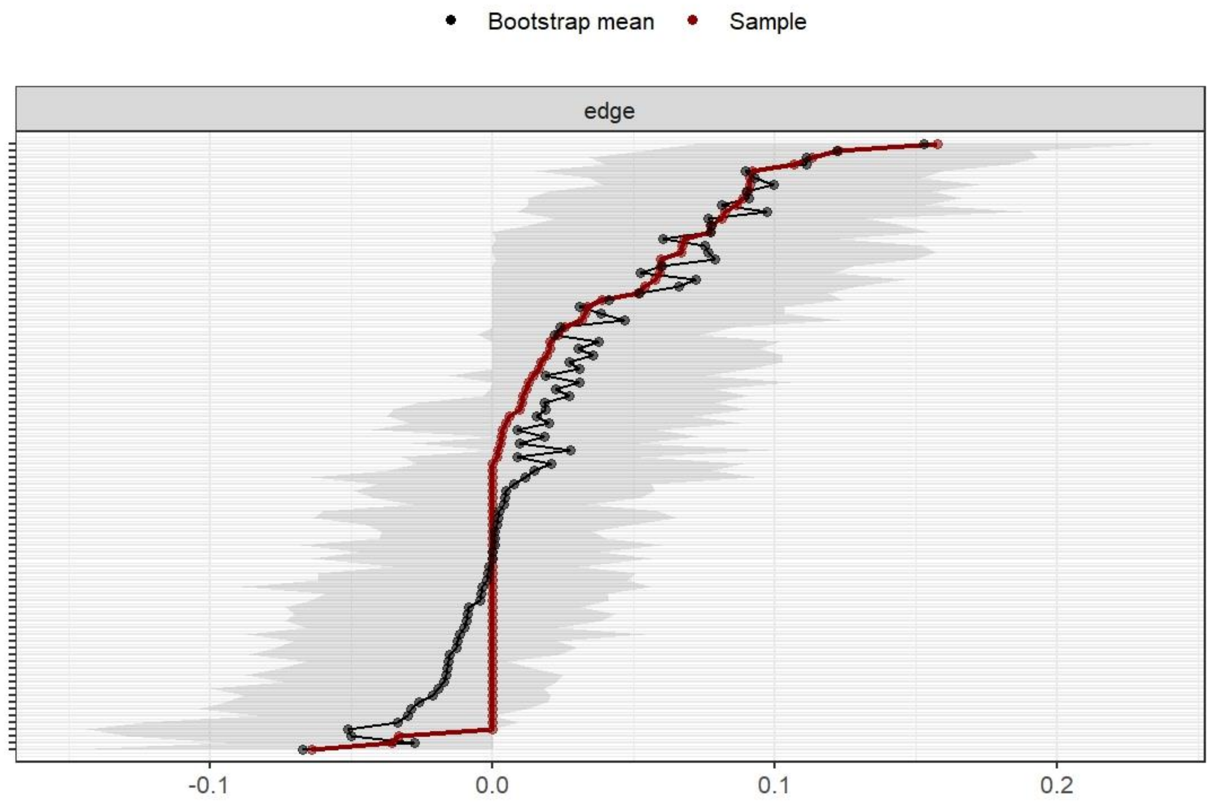

Figure S1. Edge weights accuracy for the total sample Cross-Lagged Panel Network. Note: The red line represents the edge generated by the sample. The grey indicates 95% bootstrapped confidence interval.

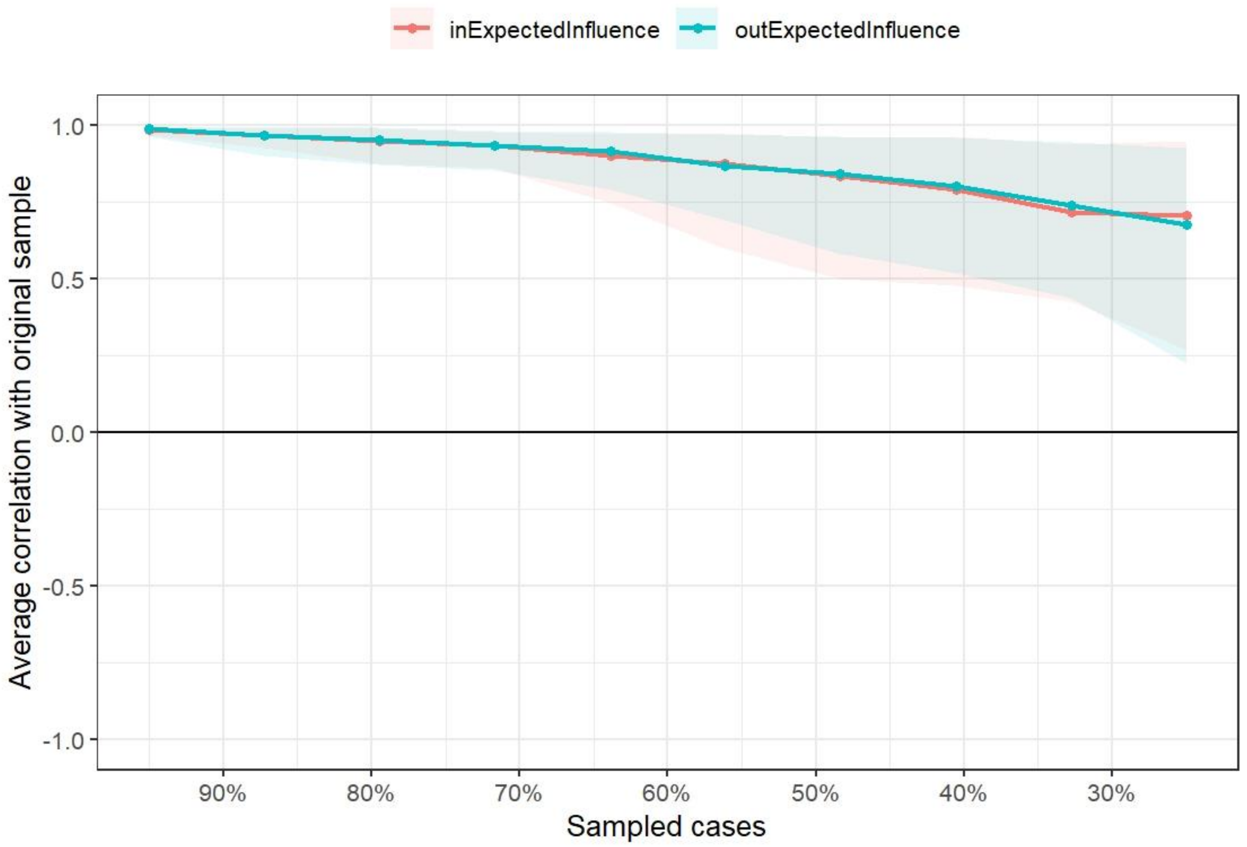

Figure S2. Centrality indices stability for the total sample Cross-Lagged Panel Network.

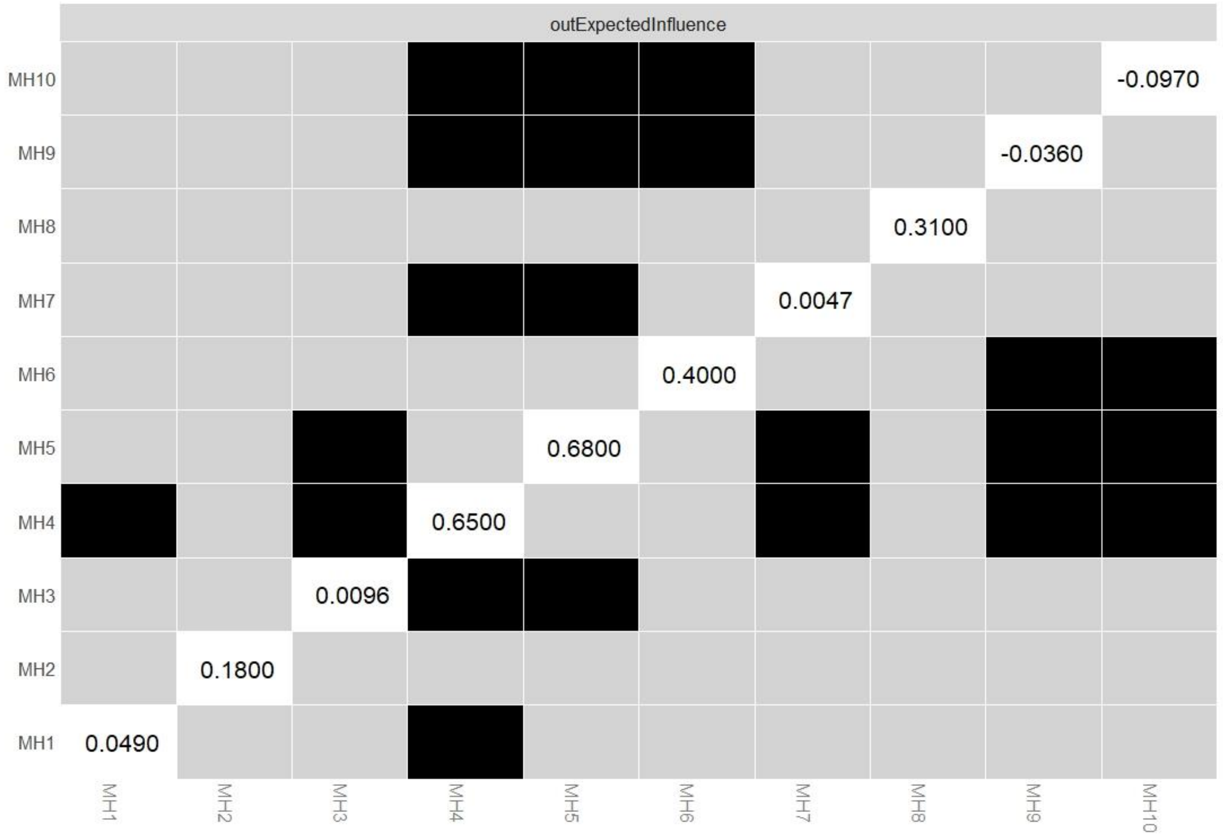

Figure S3. Centrality indices difference tests for the total sample Cross-Lagged Panel Network of out-EI. Black boxes indicate symptoms that significantly differ in centrality, and gray boxes indicate symptoms whose centrality does not significantly differ.

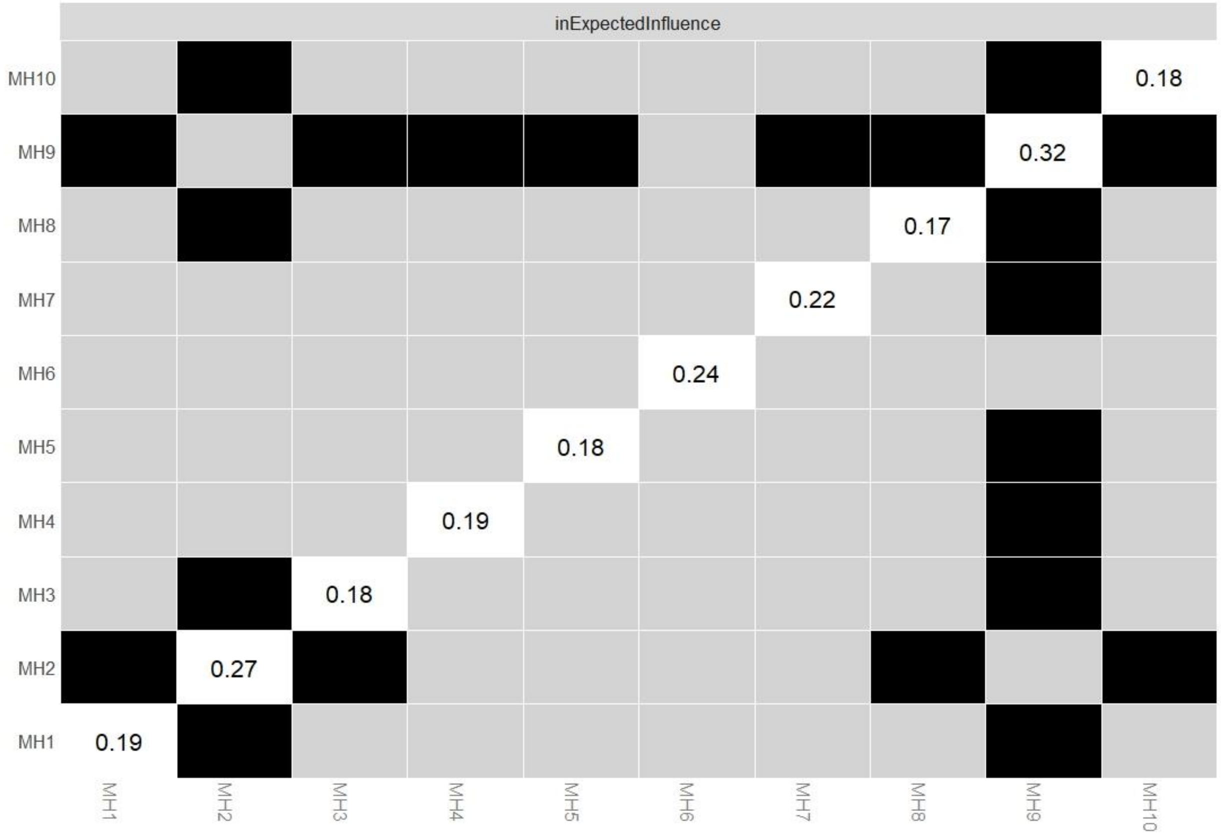

Figure S4. Centrality indices difference tests for the total sample Cross-Lagged Panel Network of in-EI. Black boxes indicate symptoms that significantly differ in centrality, and gray boxes indicate symptoms whose centrality does not significantly differ.

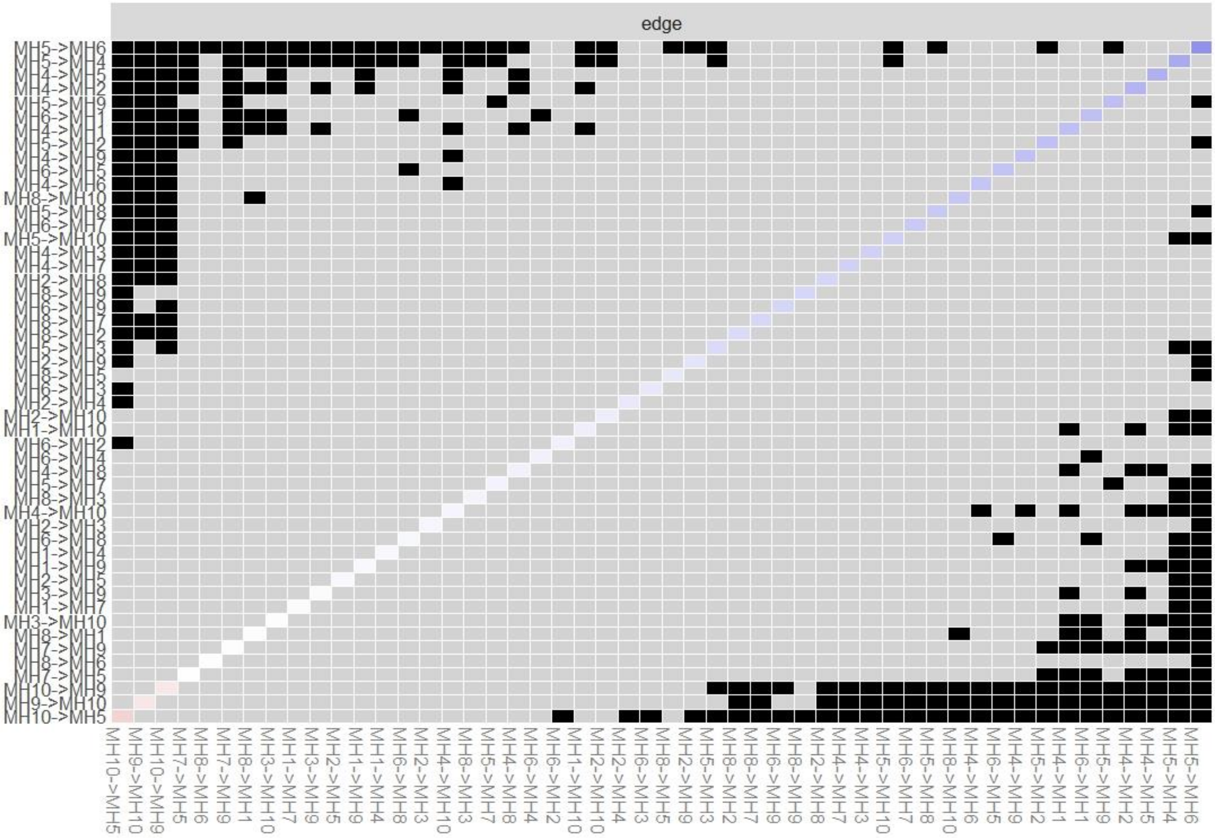

Figure S5. Edge weights difference tests for the total sample Cross-Lagged Panel Network. Black boxes indicate edges that significantly differ from each other, and gray boxes indicate edges that do not significantly differ.

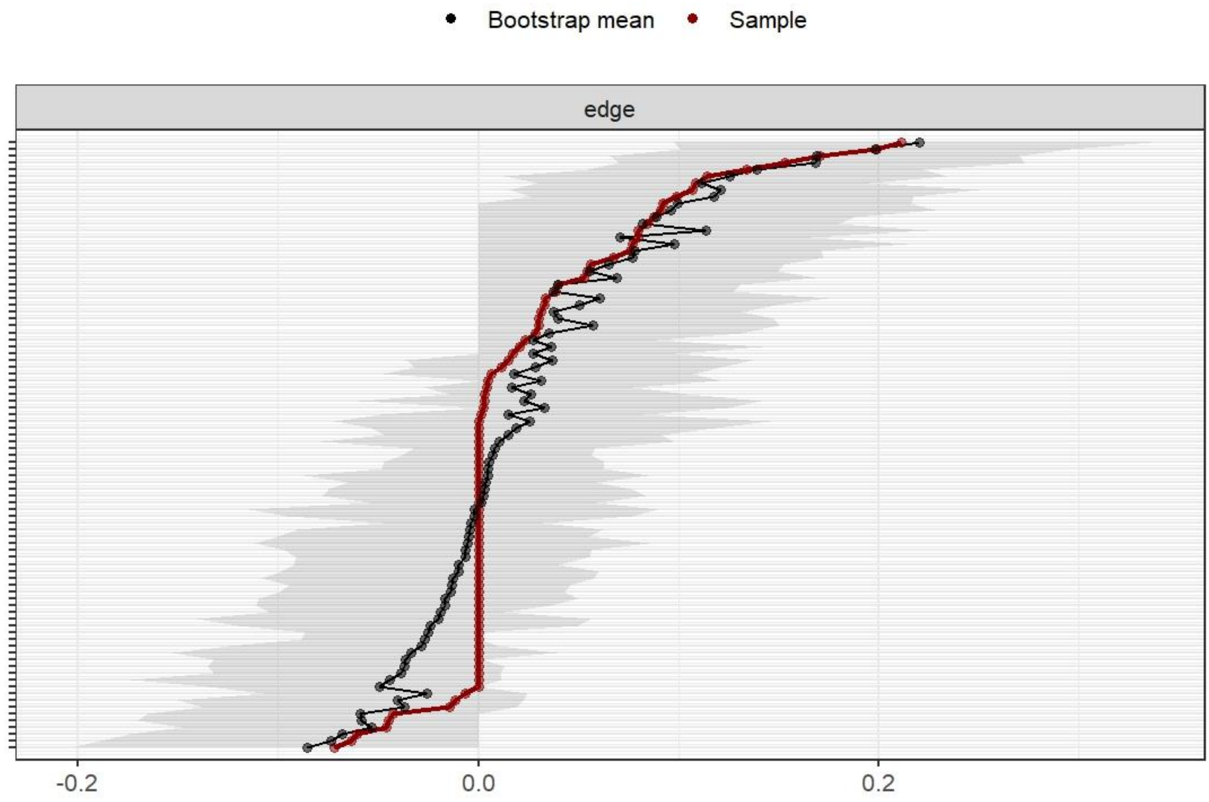

Figure S6. Edge weights accuracy for the male Cross-Lagged Panel Network. Note: The red line represents the edge generated by the sample. The grey indicates 95% bootstrapped confidence interval.

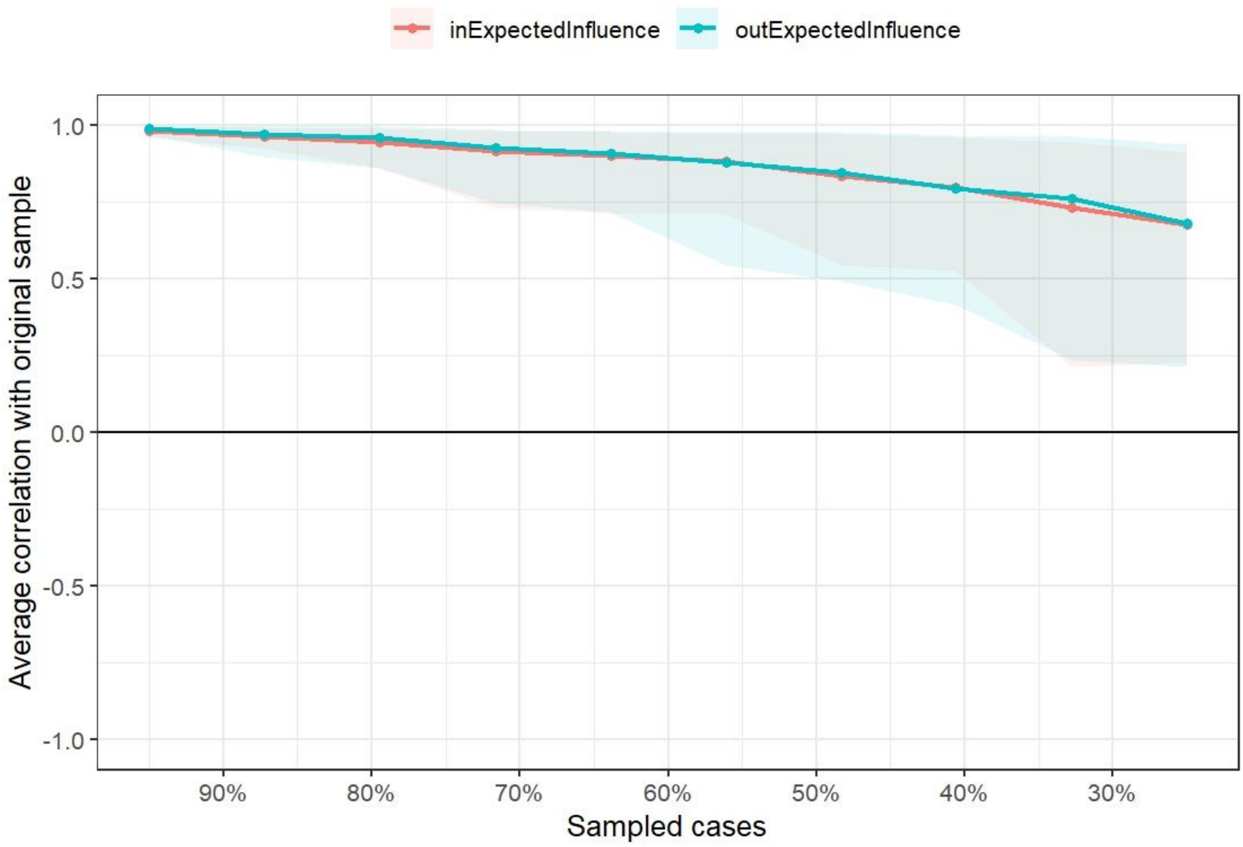

Figure S7. Centrality indices stability for the male Cross-Lagged Panel Network.

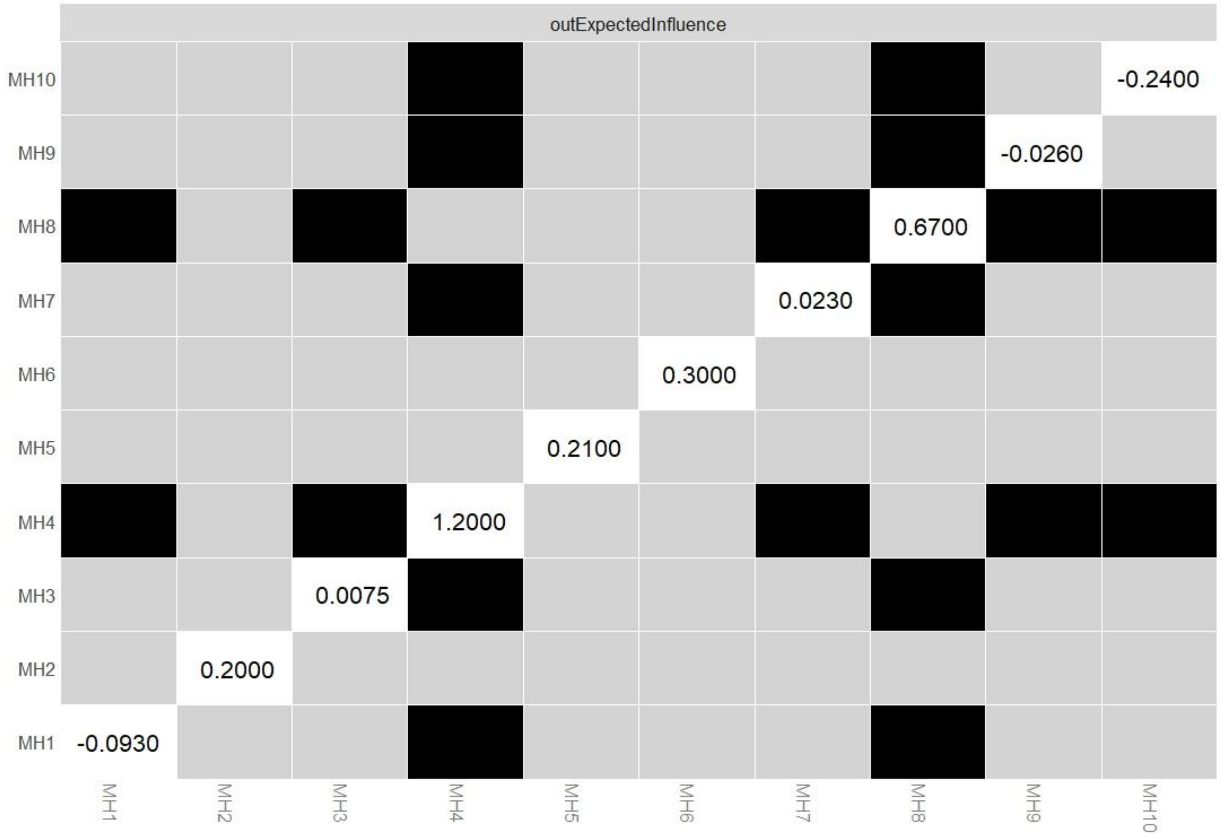

Figure S8. Centrality indices difference tests for the male Cross-Lagged Panel Network of out-EI. Black boxes indicate symptoms that significantly differ in centrality, and gray boxes indicate symptoms whose centrality does not significantly differ.

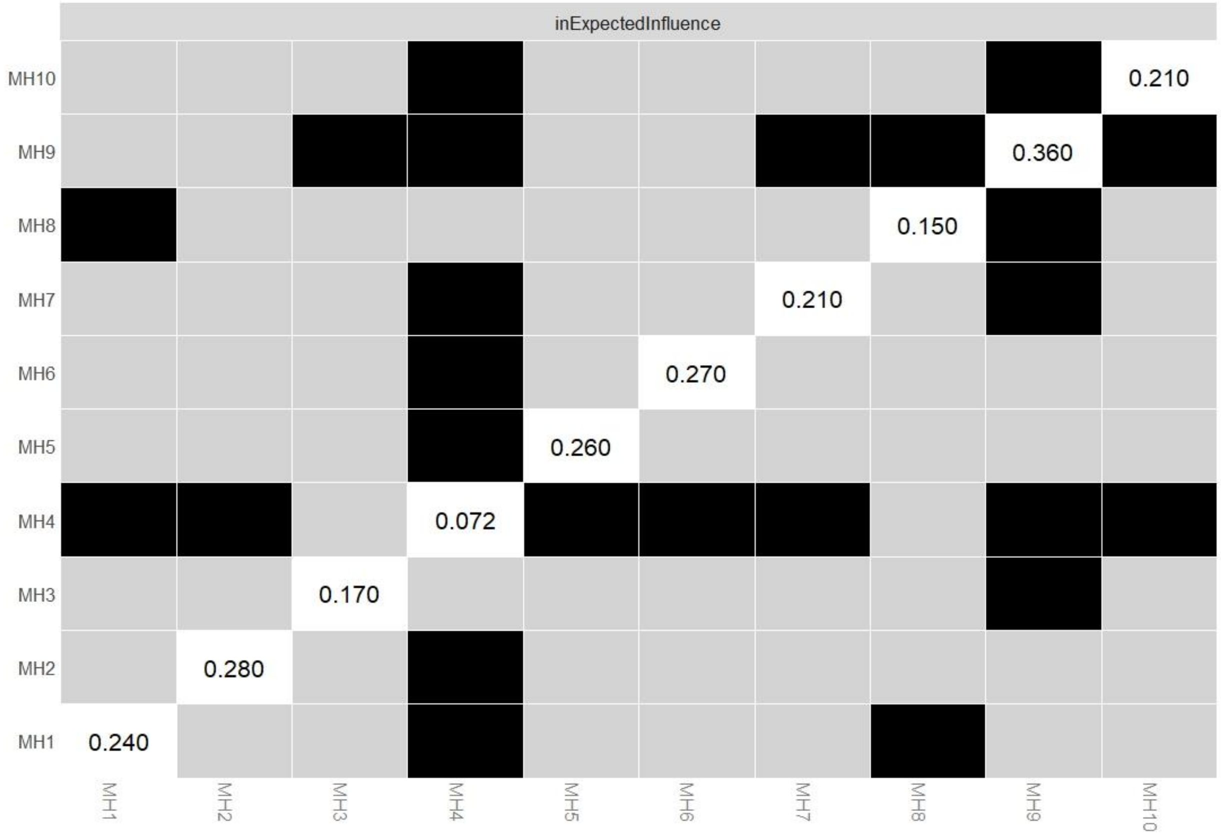

Figure S9. Centrality indices difference tests for the male Cross-Lagged Panel Network of in-EI. Black boxes indicate symptoms that significantly differ in centrality, and gray boxes indicate symptoms whose centrality does not significantly differ.

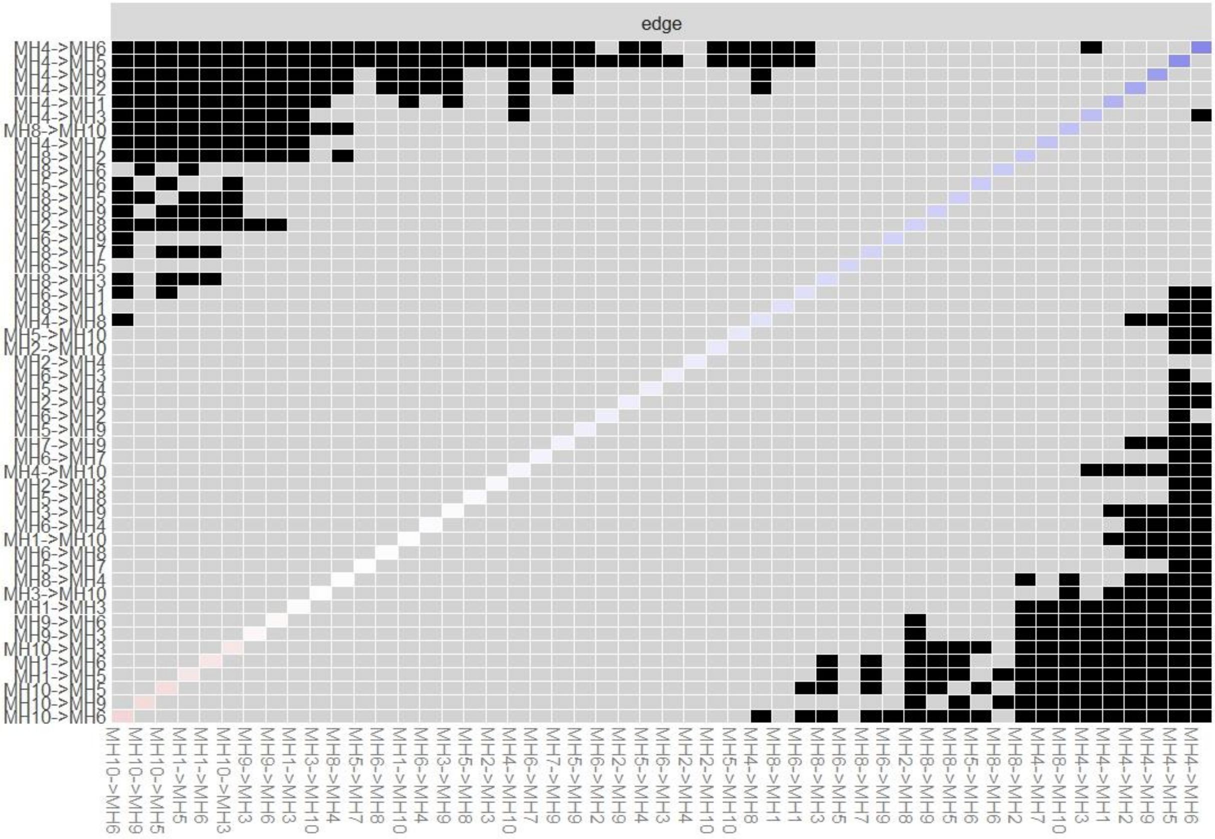

Figure S10. Edge weights difference tests for the male Cross-Lagged Panel Network. Black boxes indicate edges that significantly differ from each other, and gray boxes indicate edges that do not significantly differ.

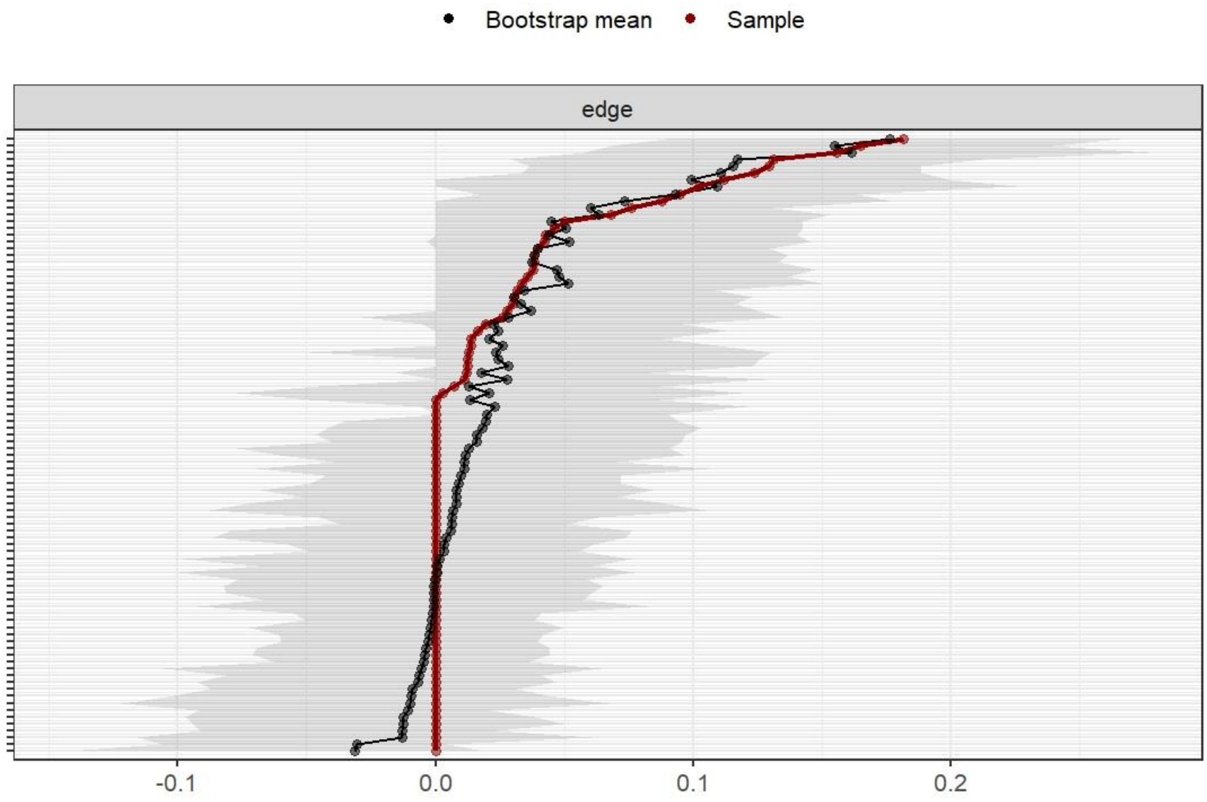

Figure S11. Edge weights accuracy for the female Cross-Lagged Panel Network. Note: The red line represents the edge generated by the sample. The grey indicates 95% bootstrapped confidence interval.

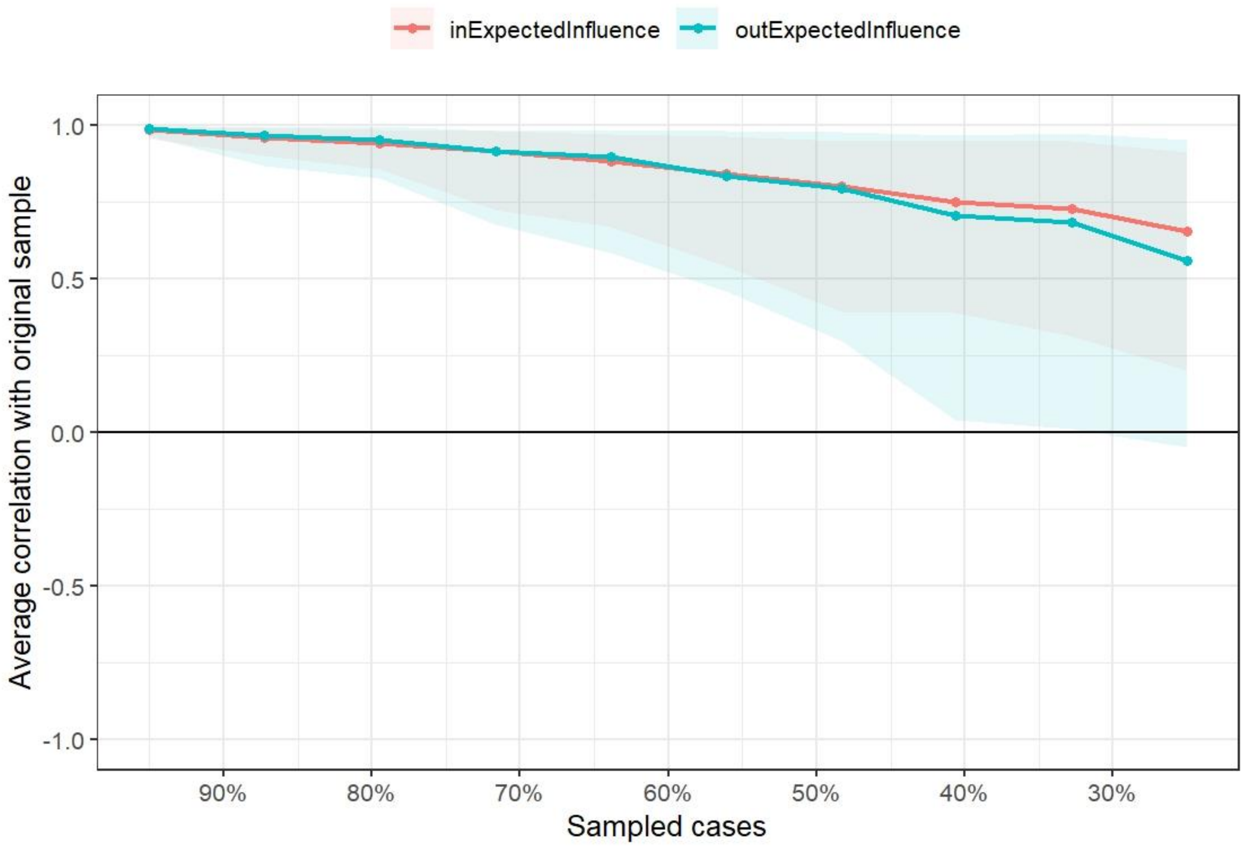

Figure S12. Centrality indices stability for the female Cross-Lagged Panel Network.

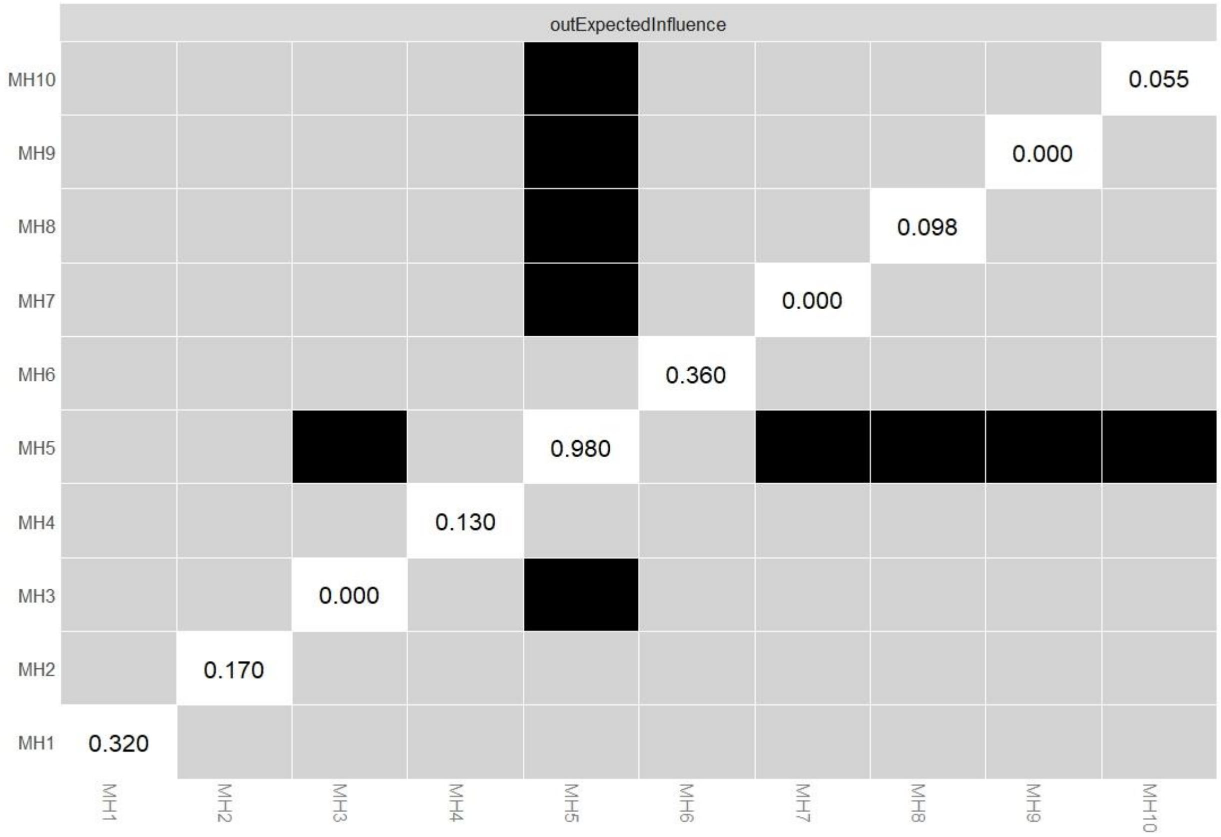

Figure S13. Centrality indices difference tests for the female Cross-Lagged Panel Network of out-EI. Black boxes indicate symptoms that significantly differ in centrality, and gray boxes indicate symptoms whose centrality does not significantly differ.

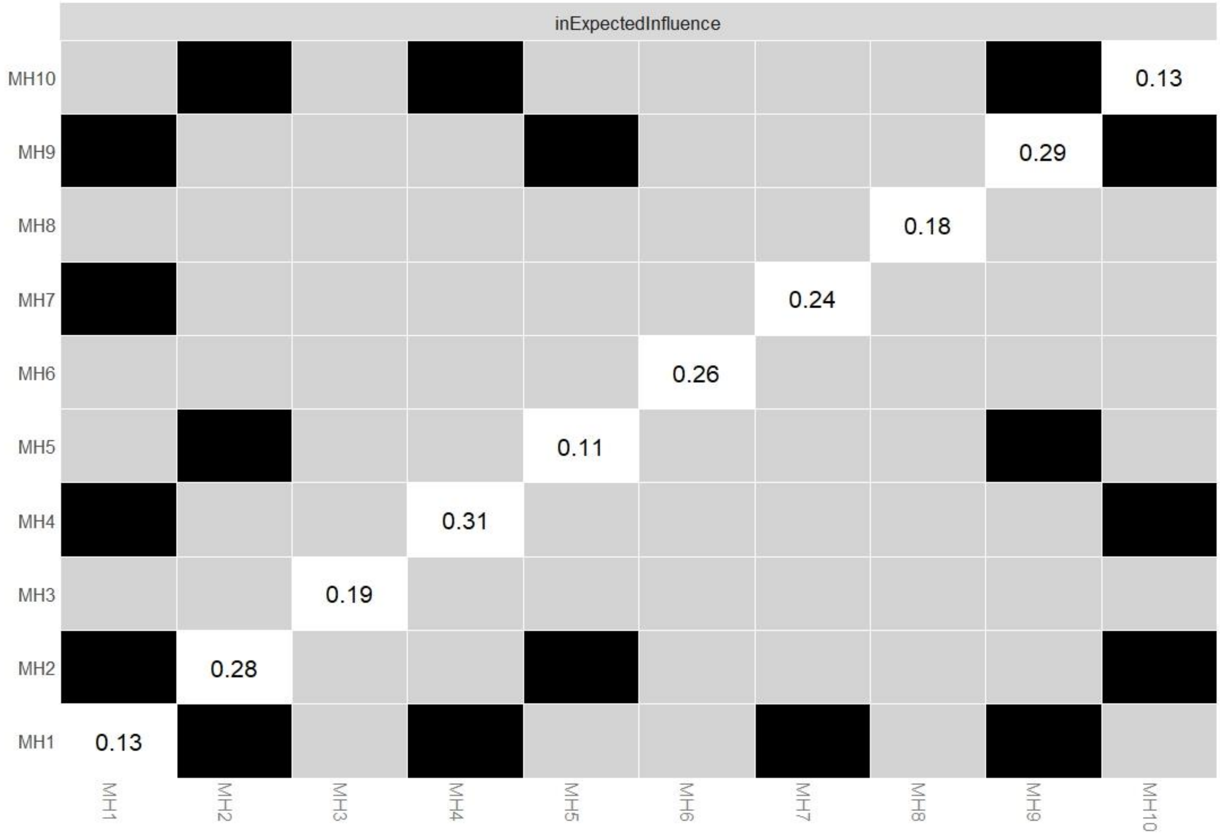

Figure S14. Centrality indices difference tests for the female Cross-Lagged Panel Network of in-EI. Black boxes indicate symptoms that significantly differ in centrality, and gray boxes indicate symptoms whose centrality does not significantly differ.

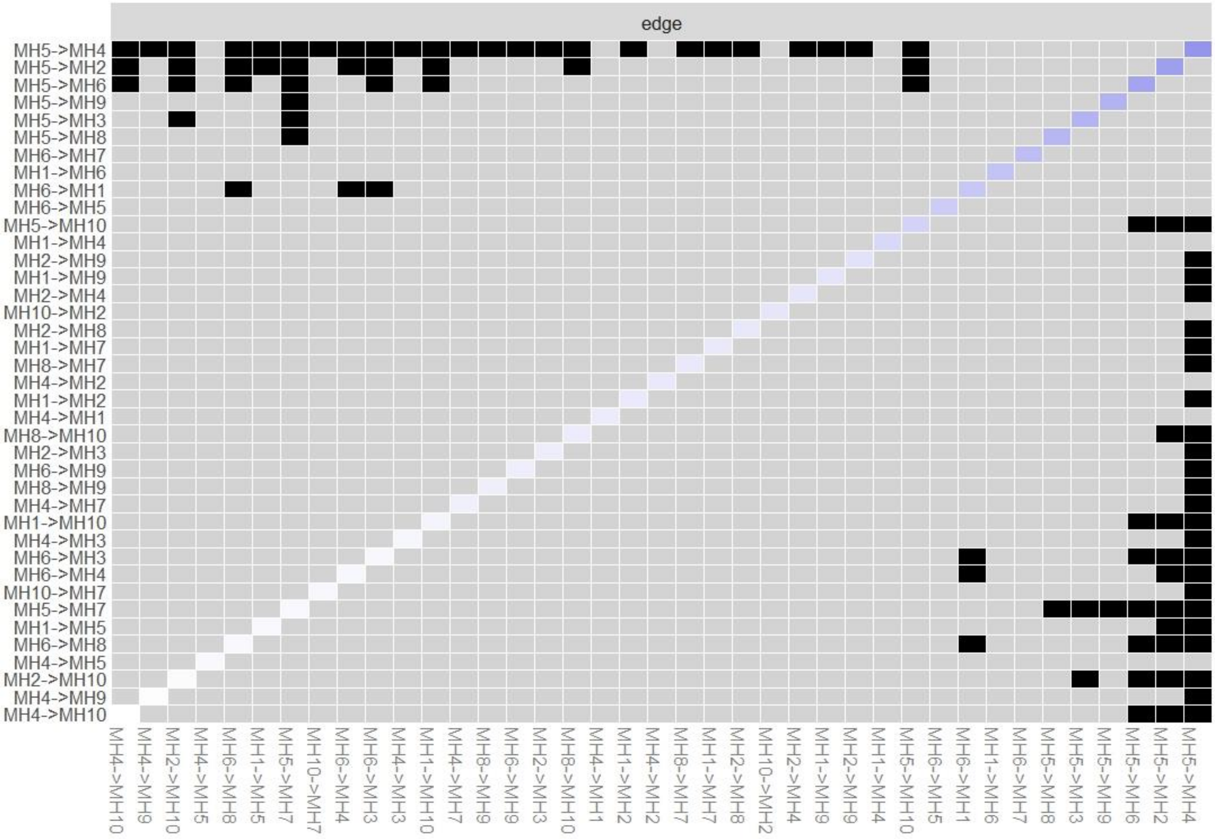

Figure S15. Edge weights difference tests for the female Cross-Lagged Panel Network. Black boxes indicate edges that significantly differ from each other, and gray boxes indicate edges that do not significantly differ.

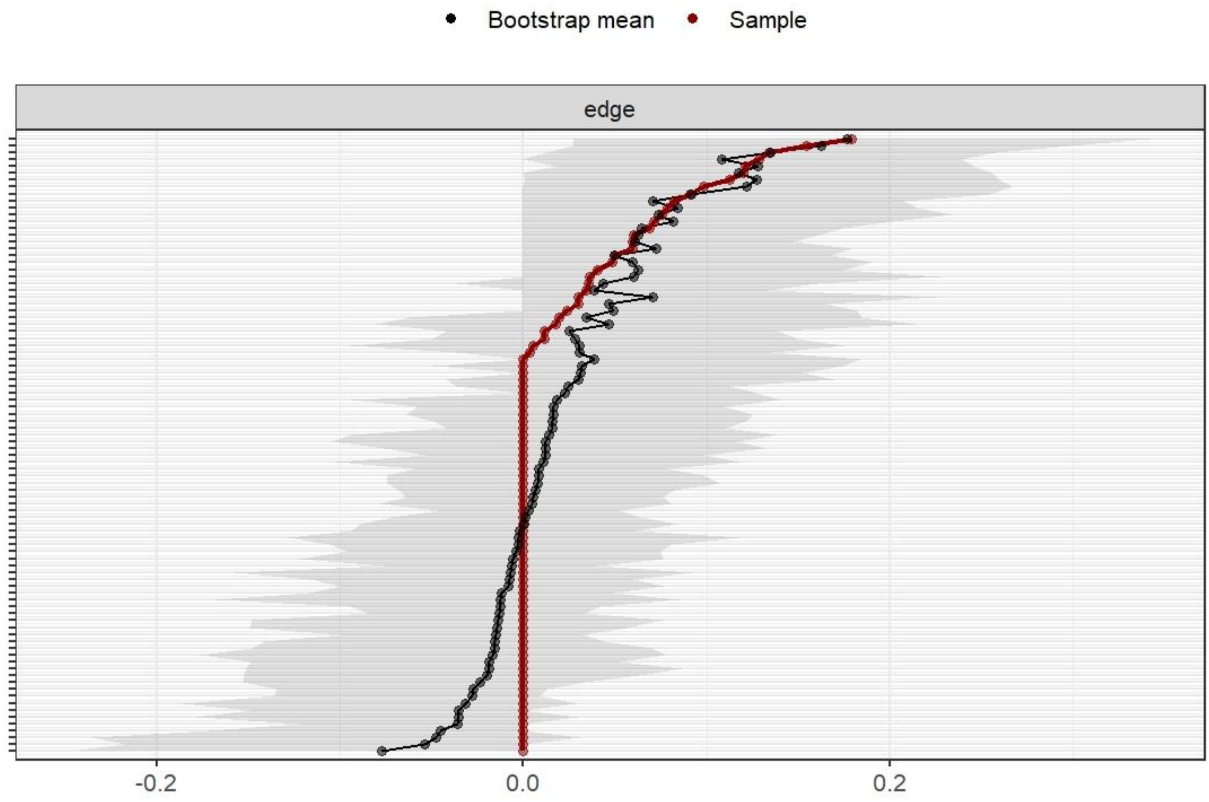

Figure S16. Edge weights accuracy for the junior high school Cross-Lagged Panel Network. Note: The red line represents the edge generated by the sample. The grey indicates 95% bootstrapped confidence interval.

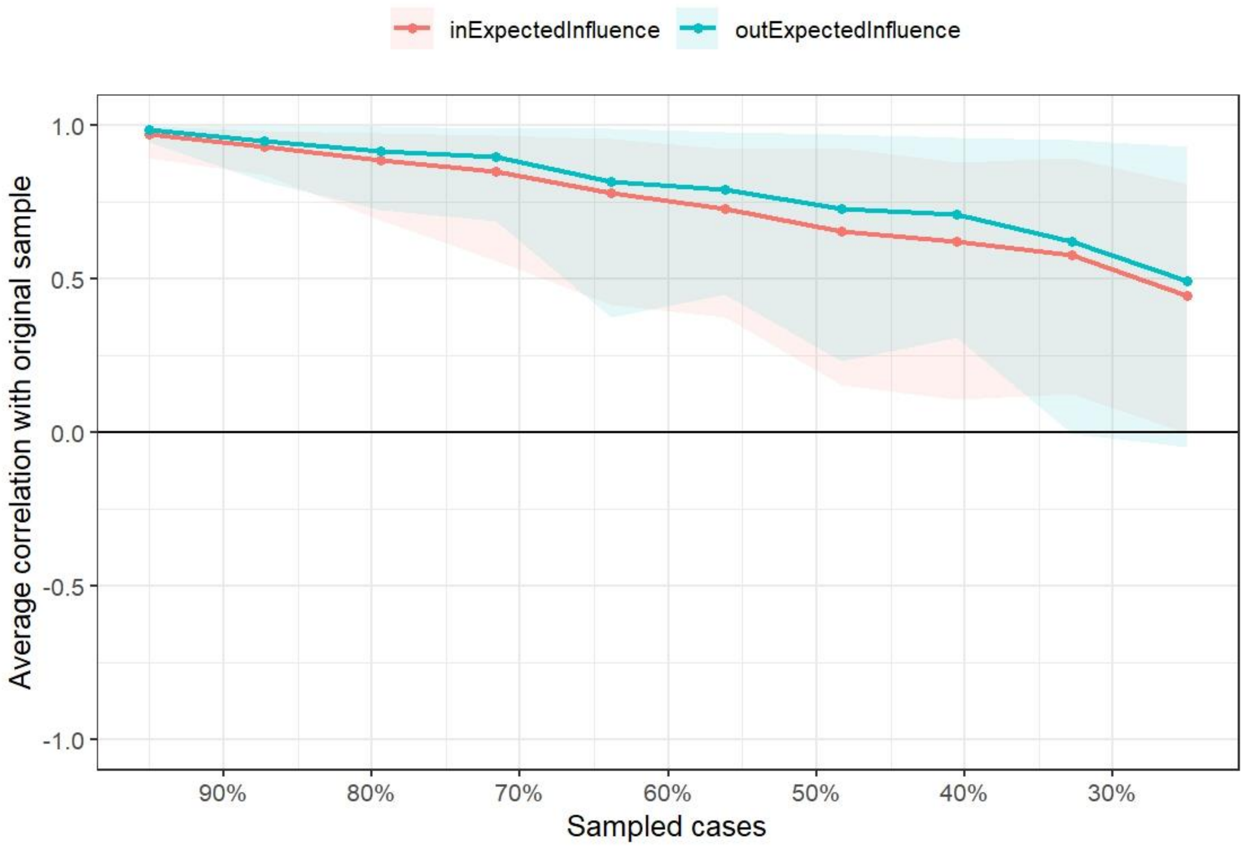

Figure S17. Centrality indices stability for the junior high school Cross-Lagged Panel Network.

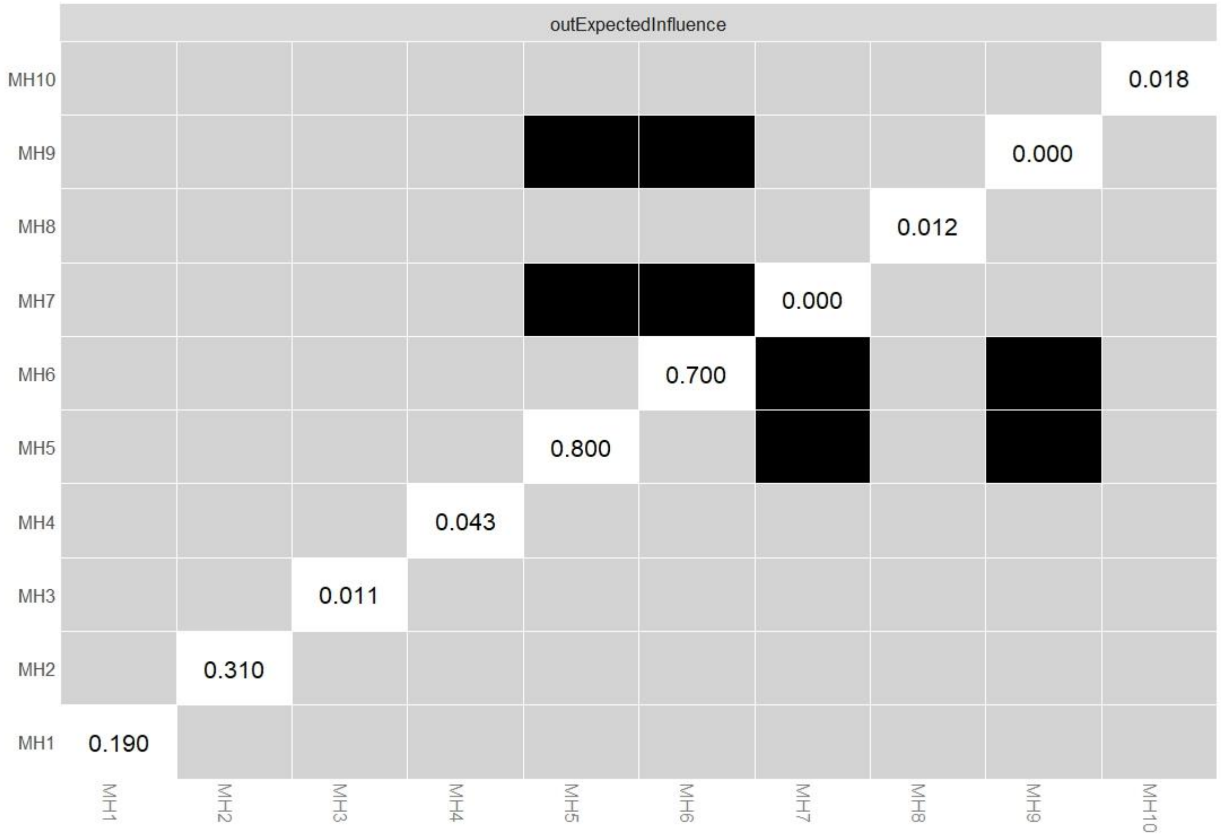

Figure S18. Centrality indices difference tests for the junior high school Cross-Lagged Panel Network of out-EI. Black boxes indicate symptoms that significantly differ in centrality, and gray boxes indicate symptoms whose centrality does not significantly differ.

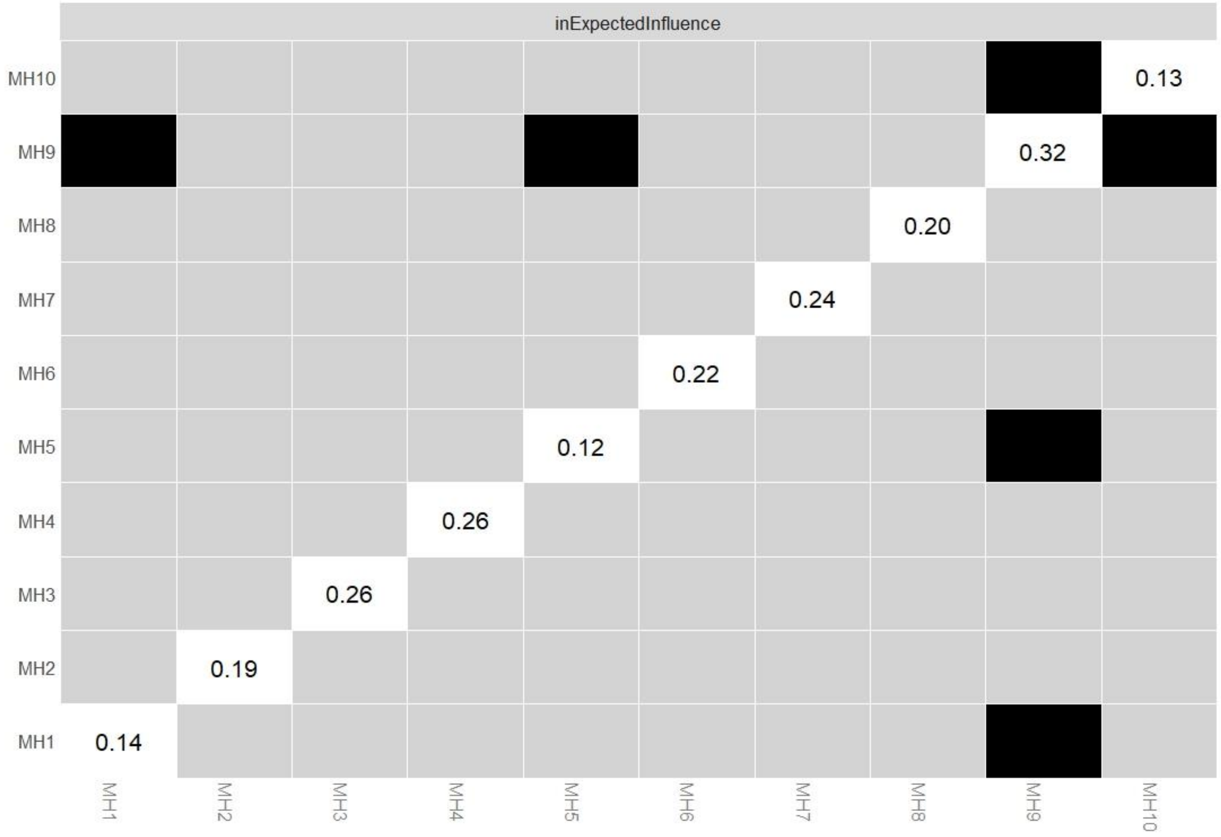

Figure S19. Centrality indices difference tests for the junior high school Cross-Lagged Panel Network of in-EI. Black boxes indicate symptoms that significantly differ in centrality, and gray boxes indicate symptoms whose centrality does not significantly differ.

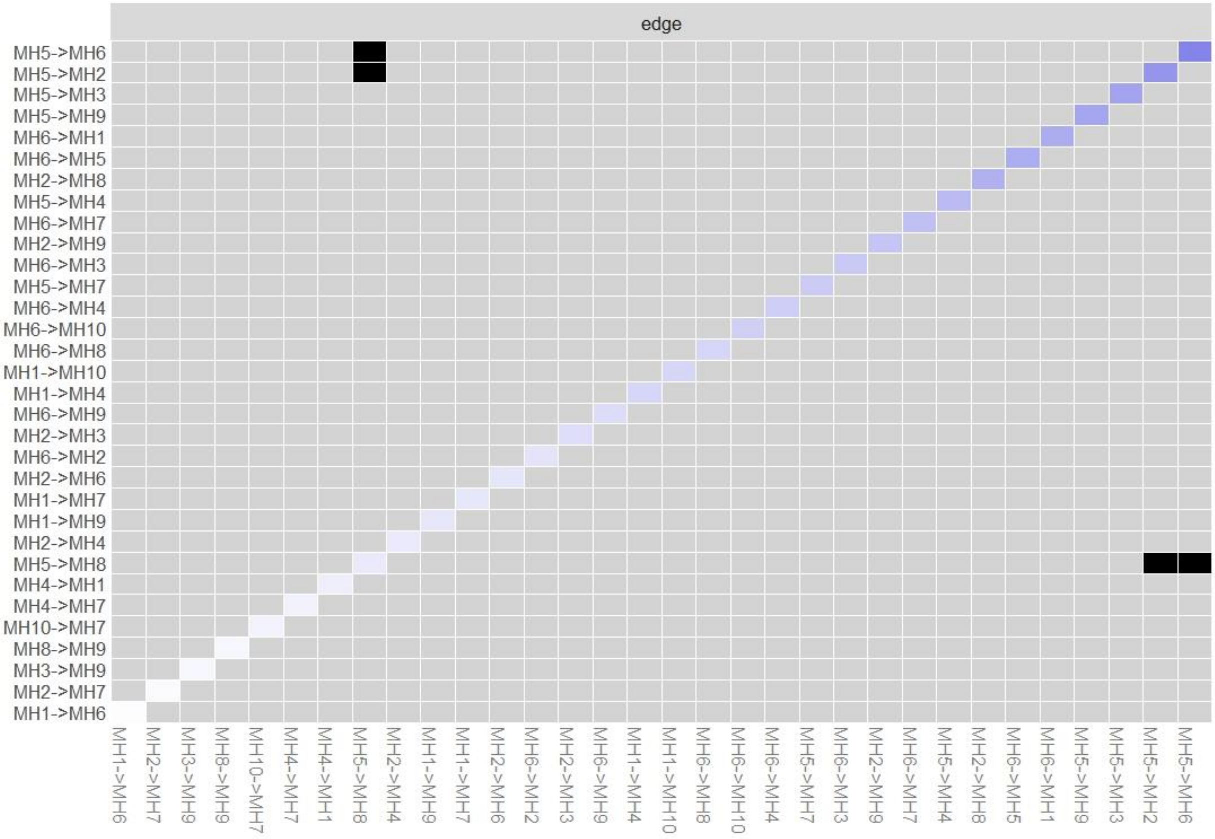

Figure S20. Edge weights difference tests for the junior high school Cross-Lagged Panel Network. Black boxes indicate edges that significantly differ from each other, and gray boxes indicate edges that do not significantly differ.

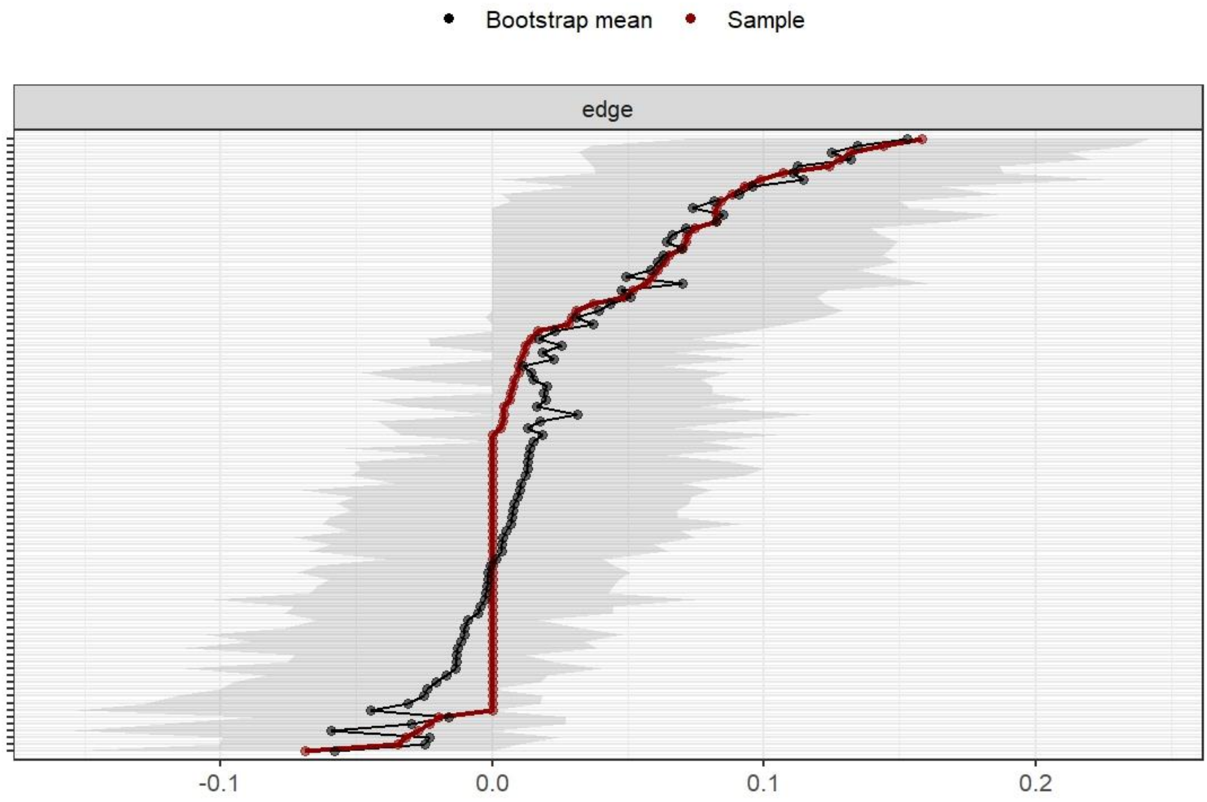

Figure S21. Edge weights accuracy for the senior high school Cross-Lagged Panel Network. Note: The red line represents the edge generated by the sample. The grey indicates 95% bootstrapped confidence interval.

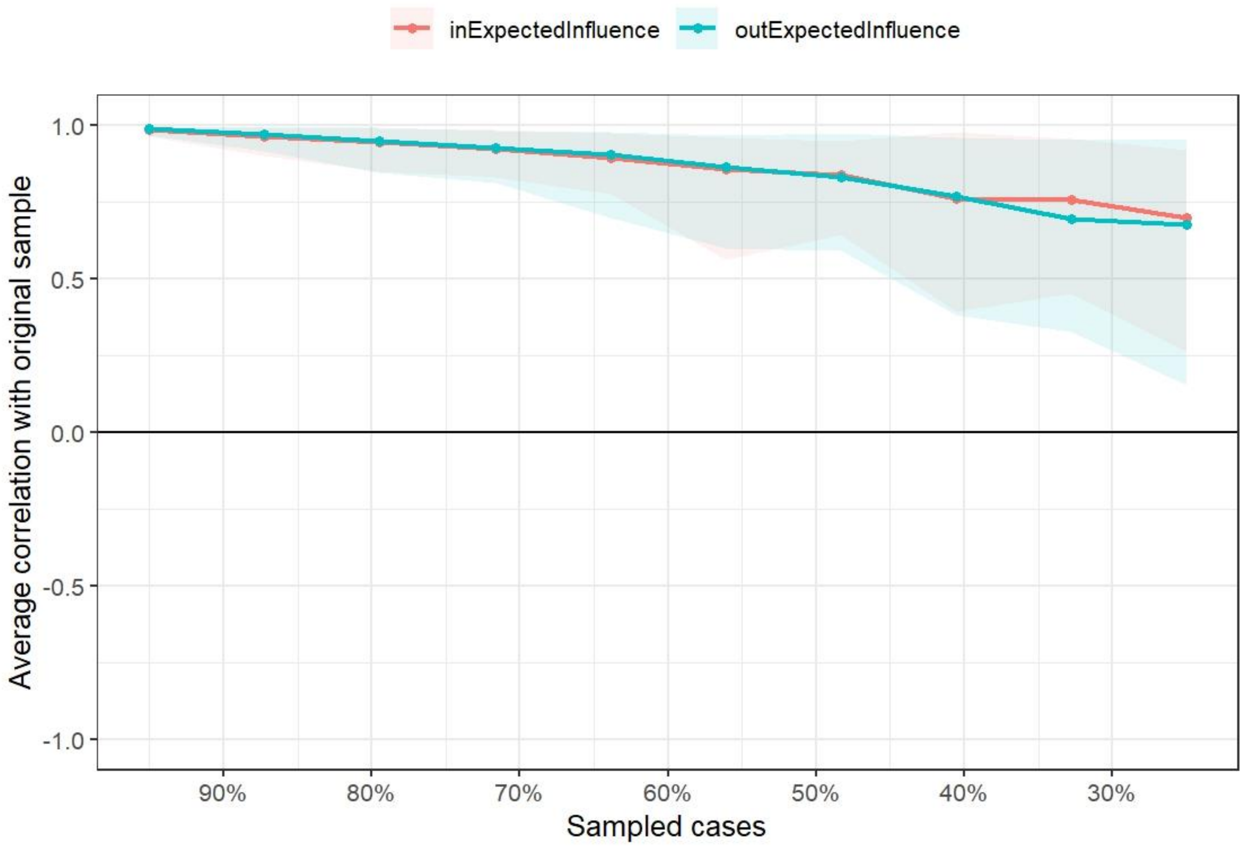

Figure S22. Centrality indices stability for the senior high school Cross-Lagged Panel Network.

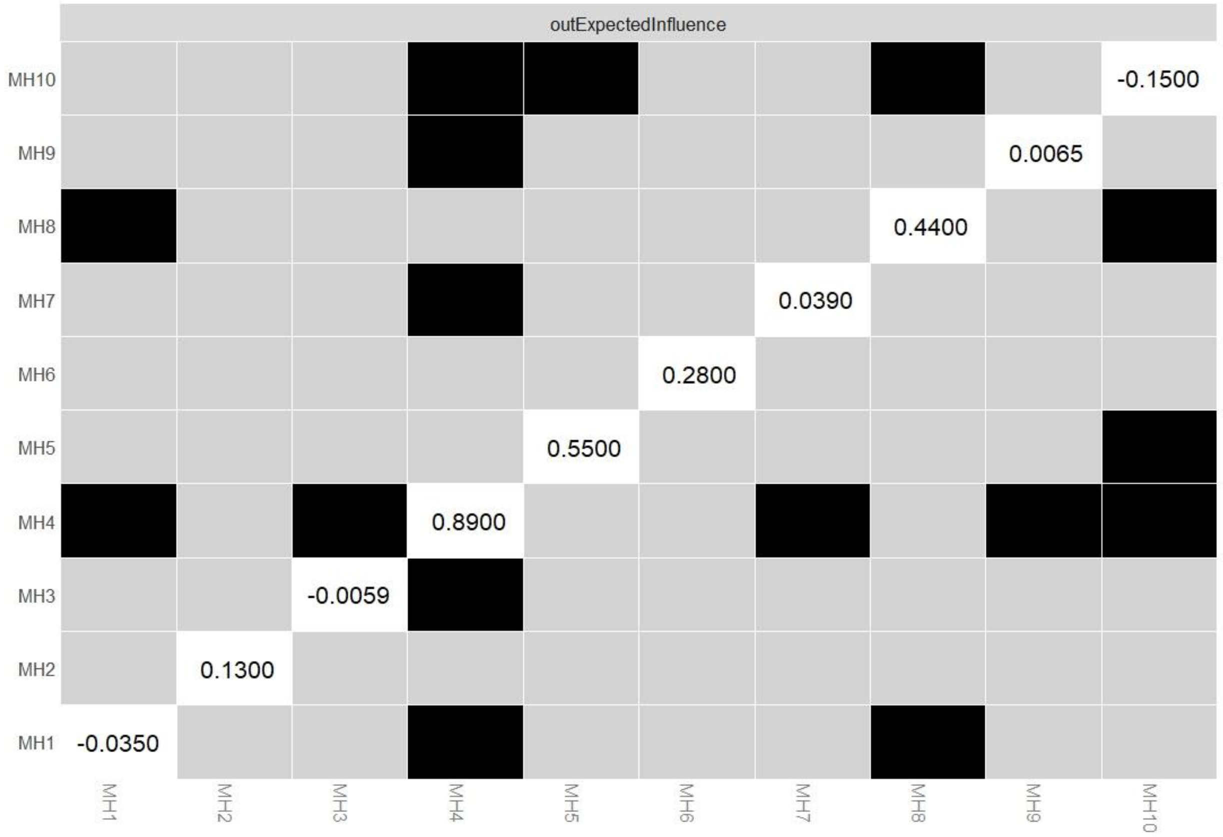

Figure S23. Centrality indices difference tests for the senior high school Cross-Lagged Panel Network of out-EI. Black boxes indicate symptoms that significantly differ in centrality, and gray boxes indicate symptoms whose centrality does not significantly differ.

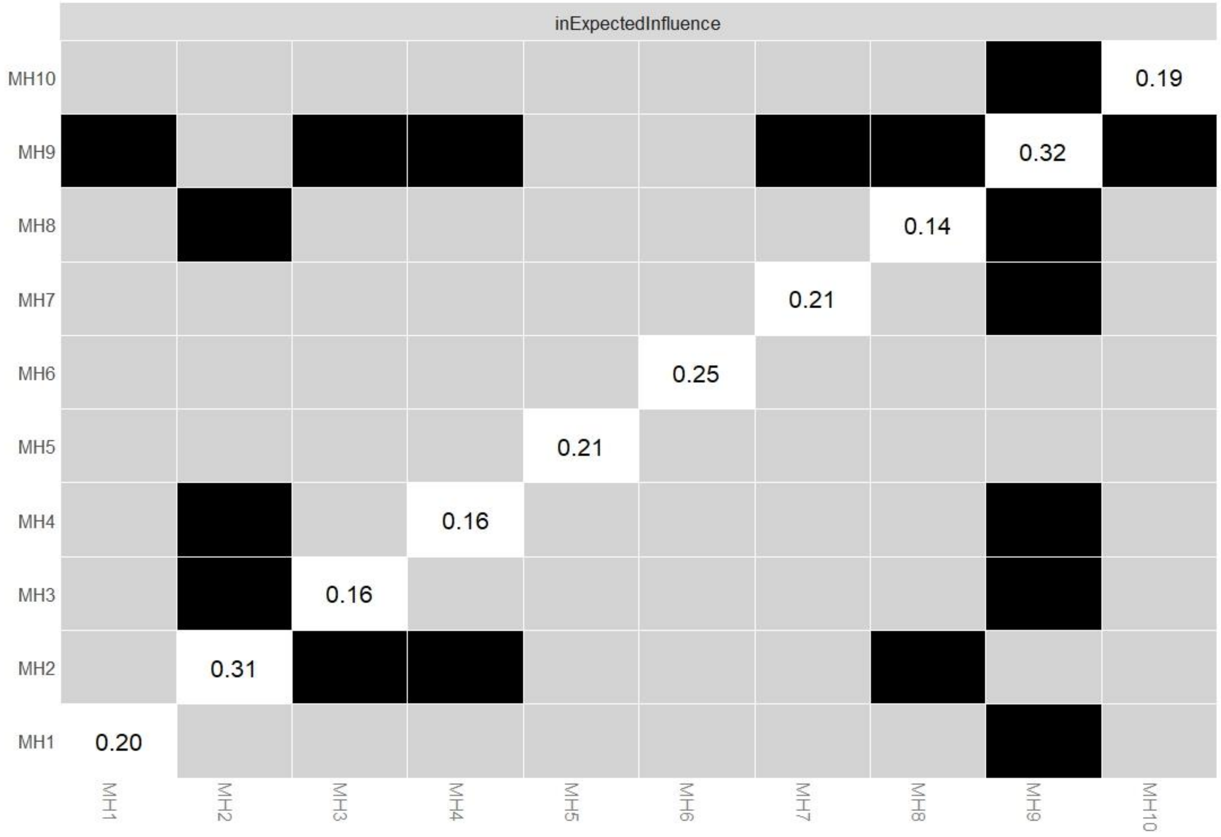

Figure S24. Centrality indices difference tests for the senior high school Cross-Lagged Panel Network of in-EI. Black boxes indicate symptoms that significantly differ in centrality, and gray boxes indicate symptoms whose centrality does not significantly differ.

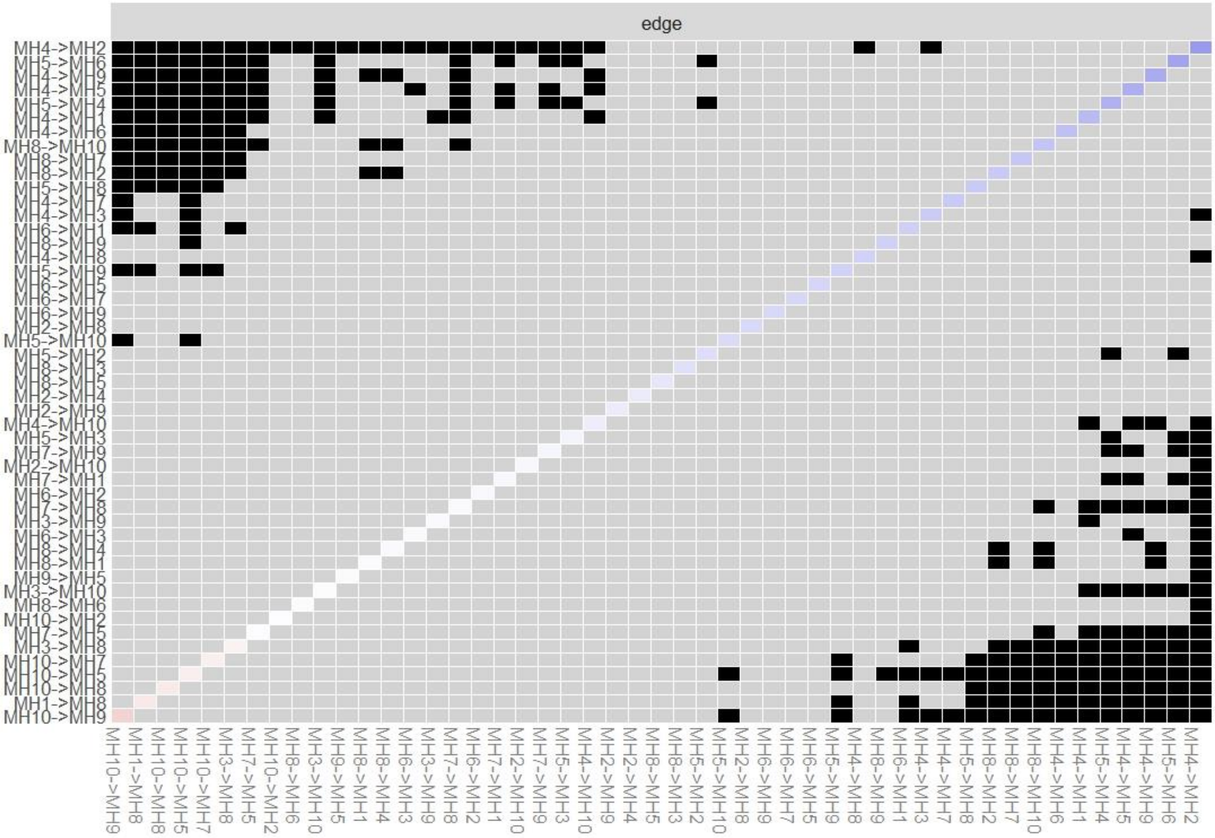

Figure S25. Edge weights difference tests for the senior high school Cross-Lagged Panel Network. Black boxes indicate edges that significantly differ from each other, and gray boxes indicate edges that do not significantly differ.
